# CDEF-Tg: Machine Learning-Based Descriptor–Embedding Fusion for Glass Transition Temperature Prediction in a Polyimide-Oriented Polymer Dataset

**DOI:** 10.3390/polym18212783

**Published:** 2026-11-07

**Authors:** Mücahit Çalışan, İbrahim Ayaz

**Affiliations:** 1 Department of Computer Engineering, Faculty of Engineering and Architecture, Bingol University, 12000 Bingol, Türkiye; 2 Department of Computer Technologies, Bitlis Eren University, 13000 Bitlis, Türkiye

**Keywords:** glass transition temperature, polymer informatics, machine learning, molecular descriptors, ChemBERTa, representation fusion, out-of-distribution evaluation, polyimide-oriented dataset

## Abstract

Predicting the glass transition temperature (Tg) of polymers from structures remains difficult, and whether descriptors and molecular-language embeddings carry complementary information is unclear. CDEF-Tg (Compact Descriptor–Embedding Fusion for Glass Transition Temperature Prediction) concatenates 205 RDKit descriptors (RDKit205) with a frozen 384-dimensional ChemBERTa embedding (ChemBERTa384) and maps the 589-dimensional vector to Tg with an ExtraTreesRegressor. It was evaluated on 10,065 identity-resolved records of a polyimide-oriented dataset under three leakage-safe protocols: IID Group-Safe (IID, independent and identically distributed), Scaffold OOD (OOD, out-of-distribution), and Similarity OOD (T = 0.90). The model was selected on validation evidence and frozen before the final-test sets were opened. In the locked final test, CDEF-Tg reached a macro mean absolute error (MAE) of 48.84 °C, versus 50.17 °C (RDKit205 alone) and 54.33 °C (ChemBERTa384 alone). Group-aware bootstrap analyses separated CDEF-Tg from RDKit205 alone in MAE only under Similarity OOD and showed no MAE advantage over the comparator RDEF-Tg. Additional validation-only controls showed a lower macro MAE for a Morgan-fingerprint partner than for CDEF-Tg (49.11 versus 53.15 °C), and ChemBERTa384 lowered LightGBM error; the ExtraTrees ordering was stable across random states (split seeds were not varied). The results suggest a modest, protocol-dependent benefit within this dataset; the additional analyses provide descriptive evidence.

## 1. Introduction

The glass transition temperature (Tg) is one of the most consequential thermal properties governing the practical behavior of amorphous and semi-crystalline polymers. Below Tg, polymer chains are kinetically frozen into a rigid, glassy state; above it, segmental mobility increases sharply, producing the rubbery or viscoelastic behavior that underlies processing operations such as extrusion, injection molding, and film casting. Tg also determines end-use performance characteristics, including stiffness, impact resistance, dimensional stability, and long-term durability under thermal cycling. Because Tg spans a wide range across polymer chemistries and is sensitive to backbone flexibility, side-group bulk, polarity, and intermolecular interactions, it functions as a key design and selection criterion in applications ranging from structural composites to packaging and biomedical materials [1,2].

Despite its importance, Tg is not trivial to determine or predict. Experimental measurement, typically via differential scanning calorimetry or dynamic mechanical analysis, is sensitive to thermal history, heating rate, sample preparation, and instrument calibration, and it is time- and resource-intensive when applied across large or combinatorial polymer libraries [3,4]. These constraints motivate structure-based prediction methods capable of estimating Tg directly from molecular composition, without requiring synthesis and physical testing of every candidate. However, purely structure-based prediction is itself difficult: Tg emerges from a combination of local chemical features and longer-range chain-level effects, and it does not always vary smoothly or monotonically with simple structural descriptors. Repeat units that appear similar by common substructure metrics can exhibit substantially different Tg values, and the relationship between molecular structure and Tg is generally non-linear, limiting the reliability of simple empirical or group-contribution rules for broad, structurally diverse polymer sets [1,5,6]. Structure-only polymer informatics is further constrained by limited data and by the dependence of experimentally reported properties on higher-order and processing-related variables that a repeat-unit representation does not encode [7,8]. Glass transition is a kinetic phenomenon, and reported glass-transition behavior is sensitive to preparation route and thermal history [3,4]. Experimental studies of molecular and orientational glasses, which are not polymers, illustrate this broad sensitivity: the glass transition of methanol cryofilms was examined as a function of added nitrogen and nitrous oxide [9], and vapor-deposited orientational glasses of the plastic crystal Freon 113 were reported to show multiple glass transitions [10]. These systems differ fundamentally from polymers, and they are cited only to illustrate the general experimental sensitivity of glass-transition behavior; none of their findings are transferred to polymer Tg prediction. Condition-dependent Tg variability of this kind is not encoded by a repeat-unit representation and may limit the accuracy attainable by structure-only models; no numerical noise floor could be established for the dataset used here.

Quantitative structure–property relationship (QSPR) methods have long been used to address this gap, and the increasing availability of machine learning (ML) regression techniques has extended their reach considerably. In this paradigm, molecules are first converted into numerical descriptors capturing physicochemical characteristics such as atom counts, topological indices, electronic properties, and functional-group information, and a regression model is then trained to map these descriptors onto the target property. Descriptor sets such as those computed by RDKit are widely used because they are interpretable, computationally inexpensive, and applicable without large labeled corpora for pretraining [1,2]. Tree-based ensemble regressors, including random forests and extremely randomized trees, have proven particularly effective for tabular descriptor-based property prediction, offering robustness to feature scaling and the ability to model non-linear interactions among descriptors without extensive manual feature engineering [1,11]. Polymer-informatics platforms and reviews have documented how descriptor- and fingerprint-based representations of repeat units support property prediction [12,13,14], and such representations have also been coupled to polymer-discovery workflows [15,16]. For Tg specifically, conventional regression and ensemble models built on descriptors or fingerprints have been reported, including systematic benchmarking of representations and algorithms and analyses of large homopolymer sets [17,18,19]. More recent studies have additionally emphasized interpretability in material-specific settings such as polyimides [1,20].

At the same time, molecular representation learning has introduced an alternative route to encoding chemical structure. Graph-based models learn structural features directly from molecular graphs and have been applied to Tg and other polymer properties [21,22]; periodic-graph representations have been proposed to reflect the repeating nature of polymers [23]; and transfer learning has been used to address data-scarce Tg prediction for specific polymer classes [24]. Pretrained molecular-language models, trained on large corpora of SMILES strings using self-supervised objectives, learn continuous embeddings that capture distributional patterns in molecular structure beyond what is explicitly encoded by hand-crafted descriptors [25,26]. ChemBERTa, a Transformer-based molecular-language model, is one such approach: it produces fixed-length embeddings that reflect learned structural and contextual regularities in molecular sequences. Polymer-specific language models such as polyBERT [27] and large-scale molecular language models such as MolFormer [28] extend this idea, and large polymer databases such as PI1M provide infrastructure for data-driven polymer modeling [29]. Representing polymers also poses challenges beyond those of small molecules, including stochastic macromolecular structure, for which dedicated line notations such as BigSMILES have been proposed [30]. Because these embeddings are derived from a fundamentally different representational mechanism than physicochemical descriptor sets, they may encode structural relationships that are not directly captured by descriptor-based features, and vice versa. Combining compact descriptors that encode explicit physicochemical information with a frozen molecular-language embedding is therefore a scientifically reasonable representation strategy to examine.

This raises a natural scientific question: whether combining a compact physicochemical descriptor set with a frozen molecular-language embedding can provide complementary predictive information for polymer Tg prediction, relative to either representation used in isolation. Such fusion is not guaranteed to be beneficial in general; the value of combining representations is likely to depend on the property being modeled, the diversity of the underlying chemical space, and the specific descriptor and embedding families involved. Rather than assuming a universal advantage, this motivates direct, protocol-controlled empirical comparison between a fused representation and its constituent single-representation counterparts.

Prior work has used physicochemical descriptors, structural fingerprints, and conventional machine learning regressors to capture structure–property trends relevant to polymer Tg prediction [1,2]. Separately, learned molecular representations, including language-model-derived embeddings, have been explored as an alternative encoding of chemical structure for property prediction tasks [25,31]. Multimodal and multi-representation fusion, for example, of language and graph representations, has likewise been explored in molecular property prediction [32,33]. However, existing evidence does not automatically establish that combining descriptor-based and learned-embedding representations yields complementary predictive value, as reported benefits of representation fusion vary across properties, datasets, and fusion strategies [31,32]. In addition, performance reported under conventional random splits does not necessarily characterize structural generalization [34,35]. Standardized, chemically or structurally motivated cross-validation protocols have been proposed in materials informatics to probe generalization more realistically [36], and quantifying similarity between polymers remains non-trivial because simple structural comparison does not capture the complexity of polymer architectures [37]. This gap motivates jointly examining descriptor–embedding fusion and leakage-safe generalization across multiple, complementary structural split regimes.

The construction of train, validation, and test partitions is central to this joint evaluation. Polymer datasets frequently contain structurally related repeat units, near-duplicate entries, or samples that share scaffolds or high pairwise similarity, and such relationships can allow structurally similar samples to appear across training and evaluation partitions, inflating apparent predictive performance and obscuring a model’s ability to generalize to structurally distinct chemistries [34,35,37]. This concern is particularly relevant for polymer property prediction, where synthetic and application interest often centers on extrapolation to novel or underrepresented structural families rather than on interpolation within a densely sampled chemical neighborhood [36,37]. Leakage-safe, group-aware evaluation protocols are therefore necessary to obtain an honest estimate of generalization performance and to distinguish genuine predictive capability from artifacts of split design.

To this end, the present study adopts three complementary evaluation protocols: an IID Group-Safe protocol, in which exact Morgan-fingerprint groups (sets of records with identical hashed circular-substructure bit vectors, defined in Section 2.2) are prevented from crossing partitions while retaining an approximately independent and identically distributed sampling regime; a Scaffold OOD protocol (OOD, out-of-distribution: held-out samples are separated from the training samples by a stated chemical criterion rather than at random) that enforces separation at the level of Murcko scaffolds [38] and exact structural groups, testing generalization to structurally distinct chemotypes; and a Similarity OOD protocol (T = 0.90) that separates connected components of structurally similar molecules at this similarity threshold, probing generalization under graded structural relatedness rather than exact scaffold boundaries. Evaluating a candidate model across all three protocols, rather than relying on a single split, provides a more complete picture of how representation choice interacts with different notions of structural generalization.

In this work, we introduce CDEF-Tg (Compact Descriptor–Embedding Fusion for Glass Transition Temperature Prediction), a framework that concatenates a compact RDKit-derived physicochemical descriptor set (RDKit205) with a frozen ChemBERTa molecular-language embedding (ChemBERTa384) into a unified representation, mapped to Tg using an ExtraTreesRegressor. The central contributions of this study are the design and evaluation of this compact early-fusion representation under a leakage-safe, group-aware evaluation framework spanning IID Group-Safe, Scaffold OOD, and Similarity OOD (T = 0.90) partitions; a model-selection procedure that relies exclusively on validation-set evidence, with the fused architecture frozen prior to any final-test evaluation; a systematic comparison of the fused representation against single-representation descriptor-only and embedding-only ablations, as well as an alternative fusion-strategy baseline, under the same locked test protocols; a paired, group-aware bootstrap analysis used to quantify uncertainty in the observed performance differences rather than relying on point estimates alone; and a descriptive analysis of how the fusion-associated performance difference varies across the observed Tg range and across evaluation protocols. The methodological novelty is deliberately modest: CDEF-Tg applies standard early concatenation and a standard tree ensemble to two existing representations, and the contribution lies in the controlled, leakage-aware evaluation of that combination rather than in a new learning algorithm. Additional analyses include control representations, a train-mean baseline, block-level permutation importance, corrected group-aware bootstrap estimation, split-structure diagnostics, a LightGBM baseline, and a fixed-split random-state analysis of ExtraTrees. These analyses were conducted after the original model selection and were not used to modify the selected model. Together, these elements are intended to provide a methodologically transparent assessment of whether descriptor–embedding fusion provides complementary predictive value for polymer Tg prediction under the evaluated protocols.

## 2. Materials and Methods

### 2.1. Dataset and Molecular Representations

The raw dataset comprised 10,066 rows, each associated with a glass transition temperature (Tg) recorded in the source dataset in degrees Celsius [39]. Molecular identity was resolved using RDKit canonical isomeric SMILES with stereochemistry preserved: records mapping to the same canonical identity were collapsed into a single identity-resolved record. Where duplicate records sharing a canonical identity carried conflicting Tg values, the Tg values within that canonical-identity group were averaged to obtain the record’s target value, and conflict metadata were retained during identity resolution. This procedure yielded the frozen dataset of 10,065 unique identity-resolved polymer samples used throughout model development and evaluation.

Dataset scope and provenance: The source file (PI_Tg_10066.csv) contains only a SMILES string and a Tg value per record; it carries no literature reference, measurement method, heating or cooling rate, or flag distinguishing experimental from computed values. The number of original literature sources, the measurement techniques, and the experimental (rather than simulated or model-generated) origin of every value could therefore not be verified in this study, and the stated Celsius unit was used without independent checking or conversion. Recorded values span 19–730 °C, with 119 records at or above 600 °C and one at or above 700 °C; they were retained unchanged because no independent basis for correcting or excluding them was available, and the sensitivity of the results to them was not assessed. The file name and the title of the cited source framework [39] refer to polyimides. A substructure analysis of the 10,065 identity-resolved structures (RDKit SMARTS [#6;R](=O)[#7;R][#6;R](=O)) found a cyclic imide ring in 9645 structures (95.8%); the screen does not show that the imide lies in the polymer backbone, and the remaining 420 structures were not further classified. The dataset is therefore described as polyimide-oriented, in the sense that its file name and source framework refer to polyimides and most structures contain a cyclic imide ring; the screen is a substructure test and not a classification of polymer backbones, and no claim is made for other polymer classes.

Identity resolution, stereochemistry, and end groups: Identity resolution reduced 10,066 raw records to 10,065 because exactly one canonical identity occurred twice. The two records had Tg values of 270.885 °C and 330.000 °C (spread 59.115 °C) and were replaced by their mean, 300.4425 °C. Stereochemistry was preserved in the canonical isomeric SMILES, so records differing only in stereochemistry remained distinct; ignoring stereochemistry, 124 groups of records shared a constitution (244 redundant rows among the 10,066 raw records relative to 9822 stereo-agnostic identities, equivalent to 243 among the 10,065 identity-resolved records), and all of them were kept as separate records. No end-group-specific processing was applied: structures were used as supplied, without neutralization, fragment selection, or salt stripping (177 multi-fragment and 14 formally charged structures were retained).

Repeat-unit attachment points: Attachment points are written as wildcard atoms (*), present in 7776 of the 10,066 raw SMILES (77.3%). They were retained as dummy atoms (atomic number 0) throughout. RDKit parses them as dummy atoms; twelve of the 217 RDKit descriptors returned non-finite values for structures containing dummy atoms (partial-charge and BCUT2D families) and were excluded, which defines RDKit205. In the Morgan fingerprint the dummy atoms enter as atoms of atomic number zero. ChemBERTa received the unmodified SMILES strings; the embedding script was configured to abort if the tokenizer emitted any unknown token, and the embeddings used here were generated with that script. How the pretrained model interprets the wildcard token was not examined, so ChemBERTa384 should be read as a general-purpose chemical-language embedding of the SMILES string rather than a model of macromolecular connectivity.

Two frozen molecular representations of different types were used throughout the study. RDKit205 encodes each polymer structure as a vector of 205 physicochemical and structural descriptors computed with RDKit [40]. Before fitting the CDEF-Tg ExtraTreesRegressor, the RDKit205 component of the fused representation was divided by a fixed constant of 16.0 and cast to 32-bit floating point (float32). This operation is a deterministic numerical-safety adapter introduced to avoid float32 overflow arising from an extremely large-magnitude descriptor value; it contains no fitted or learned parameters, performs no clipping, and deletes no descriptors. It is not a form of statistical normalization, standardization, or feature selection, and it is not presented as a methodological contribution.

ChemBERTa384 encodes each polymer SMILES string using the publicly available pretrained checkpoint DeepChem/ChemBERTa-77M-MLM, loaded with the Hugging Face AutoTokenizer and AutoModel interfaces, yielding a 384-dimensional hidden-state embedding [25]. The encoder was used strictly in a frozen, pretrained state; no task-specific fine-tuning of the underlying language model was performed at any stage of representation generation, model selection, or final evaluation. SMILES strings were supplied directly to the tokenizer without modification, and no truncation was applied; the generation pipeline enforced an effective safe sequence limit of 512 tokens. The final per-molecule representation was obtained by attention-mask-aware mean pooling over the token-level last_hidden_state outputs. No embedding normalization, scaling, PCA, feature selection, or target-informed processing was applied to the resulting ChemBERTa384 vectors. The checkpoint page does not provide a model card that documents the pretraining corpus of this specific checkpoint, and its pretraining data are therefore not asserted here. ChemBERTa-family models are transformer language models trained on SMILES strings and were introduced for molecular property prediction rather than for polymers [25]; the training configurations of other ChemBERTa variants, including ChemBERTa-2, are not assumed to be identical to those of this checkpoint. The checkpoint was used as a publicly available, general-purpose encoder with a compact 384-dimensional output. Polymer-specific encoders such as polyBERT [27] are a natural alternative. No comparison between ChemBERTa and polyBERT or any other encoder was performed, and the results therefore concern ChemBERTa384 only; they do not indicate whether a polymer-specific embedding would perform better or worse.

A Morgan (extended-connectivity) circular fingerprint representation (radius 2, 2048-bit, chirality-aware) was used separately from the two representations above, exclusively for (i) exact-duplicate grouping in the IID Group-Safe protocol, (ii) group construction in the Scaffold OOD protocol in combination with Murcko-scaffold grouping, (iii) similarity-based connected-component grouping in the Similarity OOD protocol, and (iv) as an independent compact baseline representation evaluated during validation benchmarking (Section 2.3). Morgan fingerprints were not used as an input representation for the proposed CDEF-Tg model.

**Figure 1:**
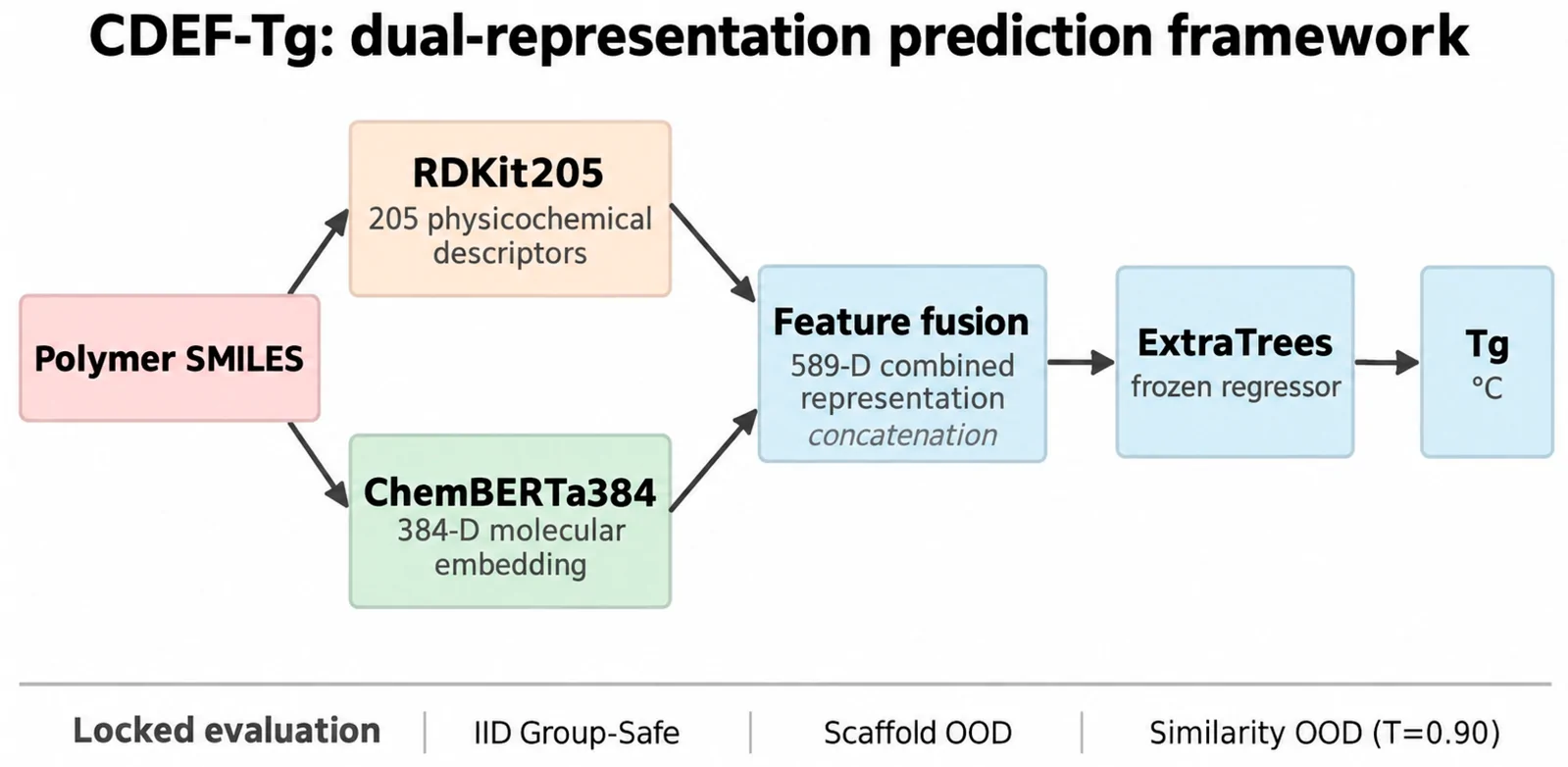
CDEF-Tg dual-representation framework for glass transition temperature prediction. Polymer SMILES are represented by 205 RDKit physicochemical descriptors (RDKit205) and a frozen 384-dimensional ChemBERTa embedding (ChemBERTa384). The two feature blocks are concatenated into a 589-dimensional representation and mapped to Tg using the frozen ExtraTrees regressor. Model selection was completed using validation-only evidence before the final-test sets were opened. Final evaluation used the locked IID Group-Safe, Scaffold OOD, and Similarity OOD (T = 0.90) protocols.

For the proposed model, RDKit205 and ChemBERTa384 were combined by early feature concatenation into a single 589-dimensional representation (205 + 384 features), preserving the fixed feature order RDKit205 followed by ChemBERTa384 (Figure 1). No learned fusion weighting, projection, or dimensionality reduction was applied at the concatenation step.

### 2.2. Leakage-Safe Evaluation Protocols

The identity-resolved dataset was partitioned into training, validation, and test subsets under three independently defined, leakage-safe evaluation protocols, each using group-aware assignment combined with global Tg rank-decile balancing and a fixed random seed (seed = 42). The three protocols (IID Group-Safe, Scaffold OOD, and Similarity OOD (T = 0.90)) differ in the chemical grouping unit used to prevent information leakage between partitions but share the same underlying dataset and target definition. Split sizes, grouping units, and additional protocol-level details are reported in Table 1.

IID Group-Safe: Groups were defined by exact identity of the 2048-bit, radius-2, chirality-aware Morgan fingerprint hash. An exact Morgan-fingerprint group is the set of records whose 2048-bit, radius-2, chirality-aware Morgan (extended-connectivity) bit vectors are identical. The fingerprint hashes circular atom-centered substructures (here, within two bonds of each atom) into 2048 bits, so identical vectors indicate identical hashed substructure content, which is broader than exact structural identity. This protocol provides the primary in-distribution comparison while still preventing leakage of identical-fingerprint inputs across partitions (train *n* = 8038, validation *n* = 1014, test *n* = 1013).

Scaffold OOD: Groups were defined as the union of non-empty Murcko-scaffold groups and exact Morgan-fingerprint groups, so that polymers sharing a Murcko scaffold or an identical fingerprint were assigned to the same partition. This protocol evaluates scaffold-level chemical generalization (train *n* = 7990, validation *n* = 1033, test *n* = 1042).

Similarity OOD (T = 0.90): Groups were defined as connected components under pairwise Morgan/Tanimoto similarity with a threshold of 0.90, so that any two polymers with Tanimoto similarity ≥ 0.90 fell within the same connected component and therefore the same partition. This protocol imposes a local chemical-neighborhood OOD evaluation, distinct in its grouping basis from the IID Group-Safe and Scaffold OOD protocols (train *n* = 8021, validation *n* = 1022, test *n* = 1022).

For all three protocols, group-aware assignment was combined with balancing across ten global Tg rank-decile bins, and partitioning used a fixed seed (42). Validation partitions were reserved exclusively for model- and hyperparameter-selection decisions; final-test evaluation was conducted only after completion of the Stage-04 model-selection and freeze procedure (Section 2.3). Group overlap between the combined development set and the test partition was zero for all three protocols (Table 1). Manuscript-facing protocol names (IID Group-Safe, Scaffold OOD, Similarity OOD (T = 0.90)) are used throughout; the corresponding internal identifiers (IID_GroupSafe, Scaffold_OOD, Similarity_OOD_T090) appear only where implementation provenance is discussed.

Global Tg rank-decile balancing is a property of the split design, not a train-only operation: the ten bins were computed over all 10,065 target values and were used only to balance the Tg distribution across partitions during group assignment, so the held-out partitions were constructed with knowledge of the label distribution of the full dataset (no model predictions were involved). Reported errors therefore refer to partitions with approximately matched Tg ranges (Table 1), and the effect of this design choice was not tested against a split without decile balancing. The Similarity OOD threshold of 0.90 was fixed at the split-design stage because lower thresholds merge the data into very large connected components; the additional component analysis supporting this choice is presented in Section 3.5, and model performance at other thresholds was not evaluated.

### 2.3. Models, Model Selection, and Final-Test Lock

A validation-only benchmark of compact, frozen baseline models was constructed during validation-only development, before final-test evaluation, using each of the three protocols described in Section 2.2 (Table 2). The baseline set comprised ExtraTreesRegressor models trained separately on RDKit205, on the Morgan fingerprint representation (2048-bit, radius 2), and on ChemBERTa384, together with a radial-basis-function support vector regressor (SVR-RBF) trained on ChemBERTa384. The three ExtraTreesRegressor baselines used identical frozen hyperparameters: 300 estimators (n_estimators = 300), a maximum feature fraction of 0.7 per split (max_features = 0.7), a minimum of one sample per leaf (min_samples_leaf = 1), a fixed random state (random_state = 42), and parallel tree construction (n_jobs = −1) [11,41]. The ChemBERTa384 SVR baseline used a train-only pipeline consisting of a StandardScaler followed by an RBF-kernel support vector regressor: StandardScaler → SVR(kernel = “rbf”, C = 10.0, epsilon = 0.1, gamma = “scale”) [41,42]. The StandardScaler was fitted on the training partition only, and its fitted parameters were applied unchanged to the corresponding validation and test partitions. No hyperparameter sweep was performed for any of the four baseline models. These baselines served exclusively to establish the validation benchmark design against which candidate architectures, including the proposed fused representation, were compared during model selection.

The proposed model, CDEF-Tg (Compact Descriptor–Embedding Fusion for Glass Transition Temperature Prediction), combines the RDKit205 and frozen ChemBERTa384 representations by early feature concatenation into the 589-dimensional fused representation described in Section 2.1, which is provided as input to a single ExtraTreesRegressor (Figure 1). The ExtraTreesRegressor was used with the following frozen hyperparameters: 300 estimators (n_estimators = 300), a maximum feature fraction of 0.7 per split (max_features = 0.7), a minimum of one sample per leaf (min_samples_leaf = 1), a fixed random state (random_state = 42), and parallel tree construction (n_jobs = −1) [11,41]. No hyperparameter search was performed for the CDEF-Tg regressor at any stage of Stage-04 model development.

To assess an alternative fusion strategy, a residual-fusion comparator, RDEF-Tg (Residual Descriptor–Embedding Fusion for Glass Transition Temperature Prediction), was evaluated alongside CDEF-Tg. RDEF-Tg first generates a primary Tg prediction using an ExtraTreesRegressor trained on RDKit205. Group-safe out-of-fold residuals from this primary predictor are then used as the regression target for a residual model, which applies the frozen ChemBERTa384 representation followed by a StandardScaler and a BayesianRidge regressor [41]. A fusion coefficient, λ, is estimated using train-only nested cross-fitting and constrained to the interval [0, 1]. The final RDEF-Tg prediction takes the form ŷ = ŷ_RDKit + λ·$\hat{r}$, where ŷ_RDKit is the RDKit205-based primary prediction and $\hat{r}$ is the residual-model prediction. RDEF-Tg is architecturally distinct from a separately defined Stage-04 candidate, ResidualFusion_Lambda1, in which λ is fixed at 1 rather than estimated from data. At the validation stage, the nested cross-fitting procedure applied to the training partition of each protocol gave λ = 1.000 for IID Group-Safe, λ = 0.947 for Scaffold OOD, and λ = 1.000 for Similarity OOD (T = 0.90). For the locked evaluation, the frozen configurations were refit on the development set of each protocol (training plus validation partitions; 9052, 9023, and 9043 records), and the RDEF-Tg λ was re-estimated within those data by the same nested procedure, without test information, giving λ = 1.000, 0.770, and 1.000, respectively; the values reported in Table 3 are these locked-evaluation values. The Scaffold OOD values of 0.947 and 0.770 therefore belong to different fitting stages. At both stages the cross-fitting groups were the atomic group identifier (IID Group-Safe), the Murcko-scaffold label (Scaffold OOD; not the union rule used to construct the split), and the similarity-cluster label (Similarity OOD (T = 0.90)). No final-test information was used to select or tune the RDEF-Tg residual-fusion mechanism.

Among the pre-specified Stage-04 candidate architectures, CDEF-Tg was selected using validation-only aggregate performance evidence across the three evaluation protocols and was frozen before final-test evaluation. MAE, RMSE, and R^2^ were the regression metrics evaluated during this validation-only comparison. Selection and freezing encompassed CDEF-Tg’s representation definitions, fusion strategy, and regressor hyperparameters. At the point of this Stage-04 freeze, no final-test predictions had been generated, no final-test metrics had been computed, and final-test information had not been used for model selection. Following the freeze, a single locked final-test evaluation was performed. No subsequent model reselection, hyperparameter tuning, feature selection, architecture replacement, or result-driven retraining was performed at any point after this locked evaluation.

The four pre-specified Stage-04 candidates were CDEF-Tg (early-fusion ExtraTreesRegressor on RDKit205 + ChemBERTa384), RDEF-Tg, ResidualFusion_Lambda1 (residual fusion with λ fixed at 1), and the frozen RDKit205-only ExtraTrees baseline. Their per-protocol and macro validation results, on which the selection of CDEF-Tg rested, are presented in Section 3.4. No candidate combining RDKit205 with Morgan fingerprints, no random-feature control, and no train-mean baseline belonged to this pre-test candidate set; these were evaluated subsequently (Section 2.5) and cannot be treated as part of the original selection.

### 2.4. Evaluation Metrics and Statistical Analysis

Model performance was quantified using three standard regression metrics computed on Tg values in °C:

(1) MAE = (1/n) Σ|y_i_ − ŷ_i_|

(2) RMSE = √[(1/n) Σ(y_i_ − ŷ_i_)^2^]

(3) R^2^ = 1 − [Σ(y_i_ − ŷ_i_)^2^/Σ(y_i_ − ỳ)^2^]

where y_i_ and ŷ_i_ denote the dataset-recorded and predicted Tg for sample i, and ỳ is the mean recorded Tg over the evaluation set. Protocol-level metrics were computed separately for IID Group-Safe, Scaffold OOD, and Similarity OOD (T = 0.90); an unweighted macro average across the three protocols was additionally reported to summarize overall performance.

Uncertainty for paired comparisons between CDEF-Tg and each frozen comparator (RDKit205-only ablation, ChemBERTa384-only ablation, and RDEF-Tg) was estimated from the frozen Stage-05 final-test predictions using 5000 paired group-bootstrap replicates. Bootstrap resampling was group-aware, using protocol-specific group labels recovered from the frozen split manifests: an atomic/exact-group label (group_atomic; sourced from atomic_group_id or equivalent alias) for IID Group-Safe, a Murcko-scaffold label (group_scaffold; sourced from murcko_scaffold or equivalent alias) for Scaffold OOD in the original analysis, and a similarity-cluster label (group_similarity; sourced from sim090_cluster_id or equivalent alias) for Similarity OOD (T = 0.90). For Scaffold OOD, this Murcko-scaffold label is not identical to the split-construction grouping rule, which used the union of non-empty Murcko-scaffold groups and exact Morgan-fingerprint groups (Section 2.2). To correct this grouping discrepancy, the paired bootstrap was repeated using the actual split groups as resampling units (the atomic group identifier of each protocol: 549 test groups for IID Group-Safe, 71 for Scaffold OOD, and 266 for Similarity OOD (T = 0.90)), again with 5000 replicates and frozen Stage-05 predictions; no model was refit. Only these corrected protocol-level intervals are reported, and the earlier Scaffold OOD intervals are superseded. For Similarity OOD (T = 0.90), the atomic group identifier and the similarity-cluster label define identical partitions; the original and the corrected bootstrap code implement the resampling differently, and the cause of the small change in the Similarity OOD intervals between the two analyses was not established (the MAE interval against RDKit205 alone excluded zero in both).

For each protocol, letting G denote the number of unique test groups under the corresponding label, each bootstrap replicate sampled G groups with replacement from the observed test groups, and all samples belonging to each selected group were included in that replicate. No additional Tg-bin or target-value stratification was applied. The same resampled sample indices were used for CDEF-Tg and all comparators within each replicate, preserving paired model comparisons; MAE, RMSE, and R^2^ were recomputed for every model in every replicate. Protocol-specific 95% confidence intervals for the paired differences were obtained from the 2.5th and 97.5th percentiles of the resulting bootstrap distributions. Macro point estimates are unweighted means of the three protocol-level values and are retained descriptively. The three test partitions are drawn from one dataset and overlap (129 shared samples between IID Group-Safe and Scaffold OOD, 255 between IID Group-Safe and Similarity OOD (T = 0.90), 75 between Scaffold OOD and Similarity OOD (T = 0.90), and 4 in all three), whereas macro intervals obtained by resampling the protocols independently do not preserve this dependence. Such macro intervals are therefore treated as exploratory and are not interpreted as dependence-adjusted evidence; inference rests on the protocol-level intervals. The original bootstrap implementation used 5000 replicates with a master seed of 42 and deterministic protocol-specific seeds.

At the protocol level, a 95% percentile interval excluding zero was treated as evidence that the paired difference was inconsistent with zero under the specified bootstrap procedure. The intervals condition on the fitted models and the single fixed split; they do not capture variability due to model refitting or to alternative split seeds.

### 2.5. Additional Experimental Analyses

The additional analyses described in this section were conducted after completion of the original model selection and locked final-test evaluation. They used only the training and validation partitions unless otherwise specified and did not influence model selection, alter the frozen CDEF-Tg configuration, or modify the locked final-test predictions and results reported in Table 2 and Table 3.

Control representations: Two validation-only controls were trained using an ExtraTreesRegressor with the same hyperparameter settings as CDEF-Tg (300 trees, max_features = 0.7, min_samples_leaf = 1, random_state = 42, n_jobs = 4). In both controls the RDKit205 descriptors were divided by 16 and cast to 32-bit floating point, a numerical-safety adapter taken over from the frozen CDEF-Tg setup, and a check confirmed that all values remained finite. The first control concatenated the scaled RDKit205 block with the 2048-bit, radius-2 Morgan fingerprint (RDKit205 + MorganR2; 2253 features). The second concatenated the scaled RDKit205 block with 384 synthetic features (RDKit205 + Random384; 589 features). For each protocol separately, the synthetic block was generated with a NumPy default random generator initialized with seed 42: independent standard-normal values were drawn for every record and dimension and then multiplied by the standard deviation (population form) and shifted by the mean of the corresponding ChemBERTa384 dimension, both computed over the training partition of that protocol only (dimensions with a training standard deviation of 1 × 10^−12^ or less were given zero spread). Random384 therefore matches the marginal training mean and spread of each ChemBERTa384 dimension but contains no record-specific structural information, no correlation between dimensions, and no relationship to Tg. Each control was fit on the training partition and evaluated on the validation partition; the test partitions were not used. The MorganR2 control asks whether another fusion partner performs comparably, and the Random384 control asks whether the CDEF-Tg gain can be reproduced by adding features of equal dimensionality that carry no structural information. A single random draw per protocol was used, so the variability of the Random384 result across draws was not assessed.

Train-mean baseline: A constant prediction equal to the mean Tg of the training partition of each protocol was evaluated on the validation and test partitions as a descriptive benchmark; no model was fitted.

Block-level permutation importance: For each protocol, the CDEF-Tg configuration (the same ExtraTrees settings, with the RDKit205 descriptors divided by 16 and cast to 32-bit floating point and concatenated with ChemBERTa384) was refit on the training partition and evaluated on the validation partition to obtain an unpermuted MAE. All columns of one feature block (RDKit205, 205 columns; or ChemBERTa384, 384 columns) were then replaced in the validation features by the same columns after a single random permutation of the validation rows, applied identically to every column of the block. The block was thus shuffled as a unit across records while the relationships among its columns were preserved, and the other block and the target values were left unchanged. Each block was permuted three times per protocol with different permutations (random-generator seed 42,000 + 100 × protocol index + repeat number, plus 50 for the ChemBERTa384 block; protocol indices 0, 1, and 2 for IID Group-Safe, Scaffold OOD, and Similarity OOD (T = 0.90)), and ΔMAE is the permuted minus the unpermuted validation MAE averaged over the three repeats. Permutation-based variable importance was described for random forests [43]; here, it was applied at the block level to a fitted ExtraTrees model. ΔMAE measures the reliance of the fitted model on a block. It does not establish independent causal contributions and does not quantify redundancy between blocks, and no SHAP values or per-descriptor permutation importances were computed. For the RDKit205 columns only, the impurity-based importances (feature_importances_) of the refit models were ranked; these are not a measure of causal or per-descriptor predictive contribution, and the descriptors are identified by their zero-based column index in the locked RDKit205 order. Descriptor names were assigned from the locked 205-descriptor list used to compute RDKit205 and were verified against the descriptor-name record for the five columns reported.

Split diagnostics: A connected-component audit of the Morgan/Tanimoto graph at thresholds of 0.70, 0.80, 0.85, and 0.90 characterizes grouping structure only and involves no model. In addition, the maximum Morgan (radius 2, 2048-bit, chirality-aware) Tanimoto similarity between each test sample and the development (training plus validation) set was computed from the frozen split manifests.

Additional algorithmic baseline (LightGBM): A gradient-boosted decision-tree baseline [44] was trained on the training partition and evaluated on the validation partition of each protocol, for RDKit205 alone and for the 589-dimensional RDKit205 + ChemBERTa384 representation. One configuration was run (LGBMRegressor with n_estimators = 300, learning_rate = 0.05, num_leaves = 31, max_depth = −1, min_child_samples = 20, subsample = 1.0, colsample_bytree = 1.0, reg_alpha = 0.0, reg_lambda = 0.0, random_state = 42, n_jobs = 8, deterministic and column-wise histogram construction; LightGBM 4.7.0, scikit-learn 1.9.1, NumPy 2.4.6, Python 3.11); the run records document this single configuration and no hyperparameter search. In this run the inputs were kept in a 64-bit floating point to avoid overflow in explicit 32-bit conversion and therefore differ numerically from the ExtraTrees inputs (unscaled 64-bit values versus division by 16 and 32-bit conversion): the RDKit205 descriptors were used without the division by 16, the 32-bit ChemBERTa384 values were promoted to 64-bit, and no clipping, imputation, or normalization was applied. The analysis used the training and validation partitions only; no test partition was evaluated.

Random-state robustness of ExtraTrees on fixed splits: The ExtraTreesRegressor models for RDKit205 and for the CDEF-Tg representation (RDKit205 divided by 16 and cast to 32-bit floating point, concatenated with ChemBERTa384; 589 features) were refit with five random states (42, 100, 200, 300, and 400) on the original training partitions of the three protocols, with the 300 trees, max_features = 0.7, and min_samples_leaf = 1 used throughout and the split seed fixed at 42, and were evaluated on the original validation partitions. Results are summarized as mean ± sample standard deviation over the five random states. This analysis measures sensitivity to the model’s own randomness on identical partitions. It does not assess robustness to different split seeds, groupings, or data partitions, none of which were varied, and the standard deviations are not confidence intervals for generalization. No test partition was used.

Reproducibility check against Stage-04B: The random-state 42 rerun did not exactly reproduce the frozen Stage-04B validation results: macro MAE was 54.87 versus 54.82 °C for RDKit205 (difference 0.050 °C) and 53.16 versus 53.15 °C for CDEF-Tg (difference 0.009 °C), with per-protocol MAE differences of up to 0.25 °C. The reference values were traced to their Stage-04B source records, but the origin of the remaining differences was not identified. The close macro agreement for CDEF-Tg reflects per-protocol differences of opposite sign (rerun minus frozen: +0.18, −0.25, and +0.10 °C for IID Group-Safe, Scaffold OOD, and Similarity OOD (T = 0.90)); under Scaffold OOD, the CDEF-Tg advantage over RDKit205 was 0.70 °C in the frozen validation results and 1.15 °C in the rerun. The unpermuted validation MAE of the block-permutation refit (Table 6: 50.26, 59.17, and 50.02 °C) agreed with the frozen Stage-04B values, whereas the random-state-42 rerun gave 50.44, 58.92, and 50.11 °C. The difference between these two additional analysis scripts was not traced to a specific cause; differences in training-row ordering are a possible candidate but were not tested, and exact reproduction is not claimed. The frozen Stage-04B validation values and the locked Stage-05 results remained unchanged. The rerun values were usedonly to calculate the mean and standard deviation of the additional random-state analysis reported in Section 3.6.

## 3. Results

### 3.1. Validation and Locked Final-Test Performance

Model selection was performed exclusively on validation data, before final-test evaluation. Table 2 summarizes the validation-only benchmark of the frozen compact baseline representations. The comparison among the pre-specified Stage-04 candidate architectures, and the selection of CDEF-Tg among them, is documented in the upper block of Table 5 (Section 3.4). CDEF-Tg (RDKit205 + ChemBERTa384, early feature concatenation, ExtraTreesRegressor) achieved the best macro validation performance in both MAE and RMSE among these candidates. Across the three evaluation protocols, CDEF-Tg attained a macro validation MAE of 53.15 °C, a macro RMSE of 70.41 °C, and a macro R^2^ of 0.629, compared with a macro validation MAE of 54.82 °C for the frozen RDKit205-only ExtraTrees baseline. This corresponds to a 3.06% reduction in macro validation MAE relative to the baseline. The same direction of validation MAE difference was observed across all three evaluated protocols. On the basis of this validation-only evidence, CDEF-Tg was selected and frozen before final-test evaluation; no final-test predictions, metrics, or comparisons informed this decision.

**Figure 2:**
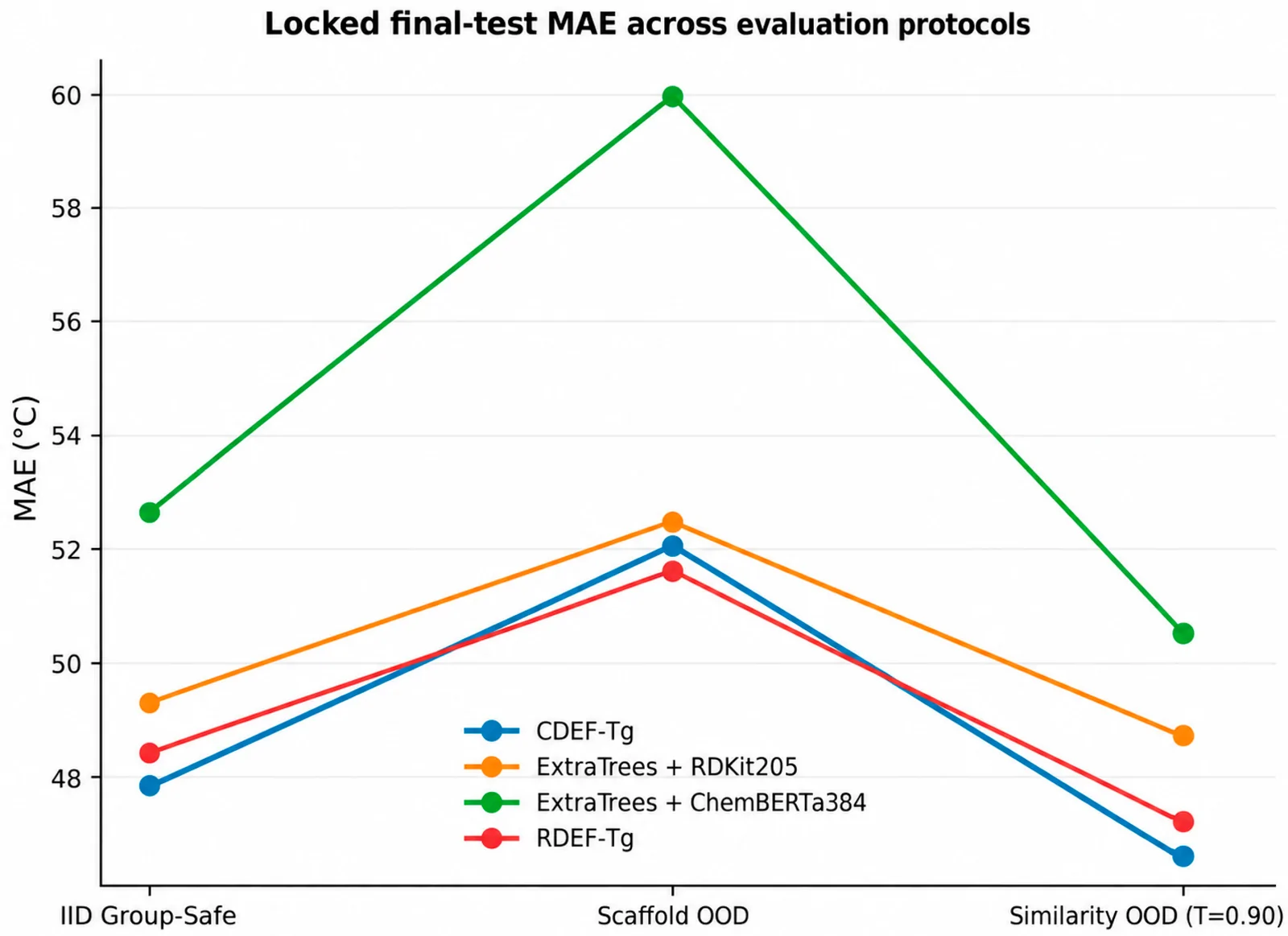
Locked final-test mean absolute error across evaluation protocols. MAE point estimates are shown for CDEF-Tg, the RDKit205-only ExtraTrees ablation, the ChemBERTa384-only ExtraTrees ablation, and RDEF-Tg under IID Group-Safe, Scaffold OOD, and Similarity OOD (T = 0.90). Lower MAE indicates lower average absolute prediction error. Statistical uncertainty and paired model differences are discussed separately in Section 3.3.

**Figure 3:**
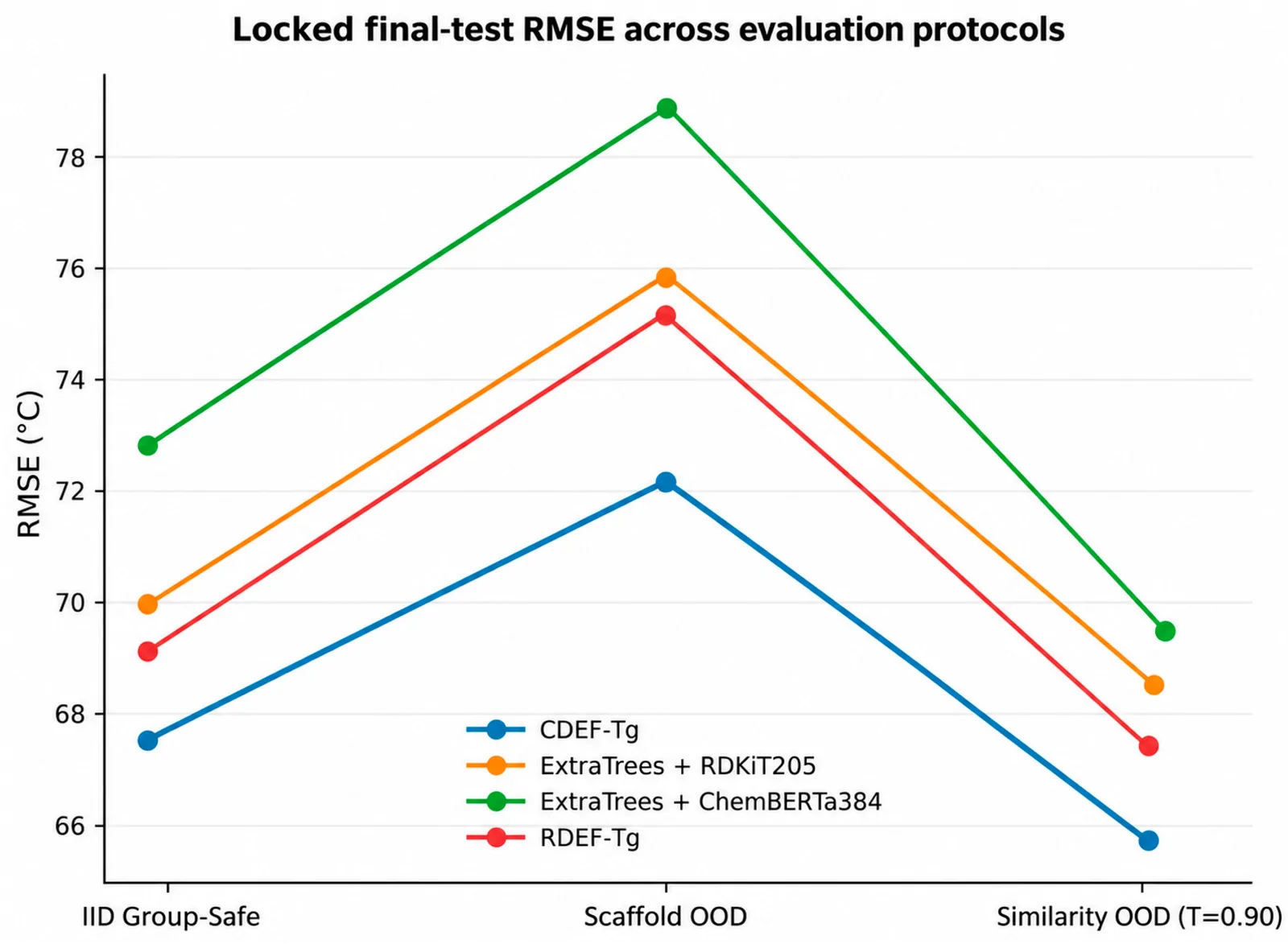
Locked final-test root mean squared error across evaluation protocols. RMSE point estimates are shown for CDEF-Tg and the three frozen comparator/ablation models under the three locked test protocols. Lower RMSE indicates better predictive performance, with greater sensitivity than MAE to large errors.

**Figure 4:**
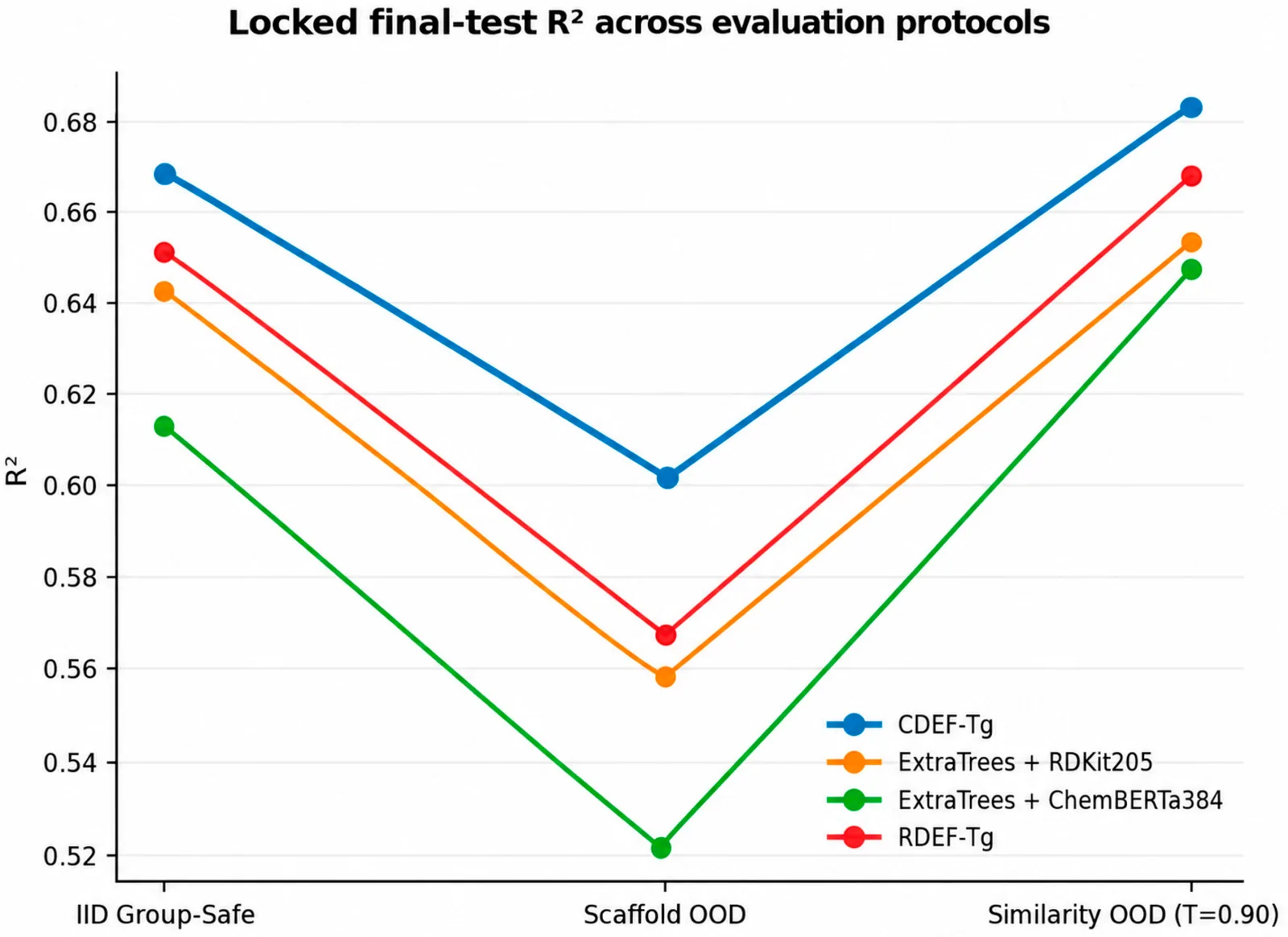
Locked final-test coefficient of determination across evaluation protocols. R^2^ point estimates are shown for CDEF-Tg and the three frozen comparator/ablation models under IID Group-Safe, Scaffold OOD, and Similarity OOD (T = 0.90). Higher R^2^ indicates a larger proportion of target variance explained by the predictions.

Following model freeze, a single locked final-test evaluation was conducted under the three protocols IID Group-Safe, Scaffold OOD, and Similarity OOD (T = 0.90). Table 3 reports the resulting metrics for CDEF-Tg together with the RDKit205-only and ChemBERTa384-only ExtraTrees ablations and the RDEF-Tg comparator; Figure 2, Figure 3 and Figure 4 display the corresponding MAE, RMSE, and R^2^ values across protocols.

Under IID Group-Safe, CDEF-Tg achieved MAE = 47.81 °C, RMSE = 67.34 °C, and R^2^ = 0.668. Under Scaffold OOD, CDEF-Tg achieved MAE = 51.98 °C, RMSE = 72.07 °C, and R^2^ = 0.602. Under Similarity OOD (T = 0.90), CDEF-Tg achieved MAE = 46.74 °C, RMSE = 65.82 °C, and R^2^ = 0.684. The unweighted three-protocol macro final-test performance of CDEF-Tg was MAE = 48.84 °C, RMSE = 68.41 °C, and R^2^ = 0.651.

Descriptively, CDEF-Tg showed lower point-estimate MAE and RMSE, and higher R^2^, than the RDKit205-only ablation (macro MAE = 50.17 °C, RMSE = 71.54 °C, R^2^ = 0.618) and the ChemBERTa384-only ablation (macro MAE = 54.33 °C, RMSE = 73.70 °C, R^2^ = 0.594) at the macro level and within each individual protocol. Relative to RDEF-Tg (macro MAE = 49.11 °C, RMSE = 70.54 °C, R^2^ = 0.629), CDEF-Tg showed a lower macro MAE point estimate, although under the Scaffold OOD protocol specifically, RDEF-Tg attained a slightly lower point-estimate MAE than CDEF-Tg (51.67 °C versus 51.98 °C). CDEF-Tg did not attain the lowest Scaffold-OOD MAE point estimate among the evaluated methods. As addressed in Section 3.3, the protocol-level MAE intervals for CDEF-Tg versus RDEF-Tg include zero, and these point-estimate comparisons should not be interpreted as indicating a distinguishable difference in the absence of that evidence. For every model and protocol, the locked test MAE was lower than the corresponding validation MAE (for example, CDEF-Tg under Scaffold OOD, 59.17 °C on validation versus 51.98 °C on test). The locked models were refit on the larger development sets, and the test partitions differ in group composition (Table 7); the contribution of these factors was not examined, and validation and test values are therefore not compared as a generalization gap.

The ChemBERTa384-only ablation had the highest MAE in every protocol, and its gap to the other models was largest under Scaffold OOD (59.96 °C versus 51.67–52.40 °C for the other three models; Figure 2). That gap was larger on the locked test partition than on the validation partition, where ChemBERTa384-only (63.06 °C) trailed RDKit205-only (59.87 °C) by about 3 °C (Table 2), and its disadvantage relative to CDEF-Tg was distinguishable from zero in every protocol (Table 4). A plausible but untested explanation is that a frozen, general-purpose chemical-language encoder that was not tuned to Tg encodes less Tg-relevant information than the RDKit205 descriptors; consistent with this, the permutation analysis (Table 6) shows that the fitted CDEF-Tg model relies more on RDKit205 than on ChemBERTa384.

### 3.2. Representation Ablation and Protocol-Dependent Behavior

Comparison of CDEF-Tg against its two single-representation ablations provides a direct empirical comparison between the fused representation and its two constituent single-representation models (Table 3; Figure 2, Figure 3 and Figure 4). Across all three locked final-test protocols, CDEF-Tg showed lower MAE and lower RMSE, and higher R^2^, than both the RDKit205-only ExtraTrees ablation and the ChemBERTa384-only ExtraTrees ablation, without exception among these three architectures. The ChemBERTa384-only ablation produced the highest MAE and RMSE and the lowest R^2^ of the four evaluated architectures in every protocol. These observations show that the fused model had lower point-estimate error than either single-representation ablation. This is compatible with the two representations carrying partly different predictive information, but it does not by itself establish complementarity or a mechanistic basis for it. This interpretation is further limited by the additional analyses: the benefit of adding ChemBERTa384 to RDKit205 is small and is not consistently distinguishable from zero at the protocol level (Section 3.3), and an alternative partner (the Morgan fingerprint) gave a lower validation MAE (Section 3.4). Complementarity between the two representation families is therefore not established.

**Figure 5:**
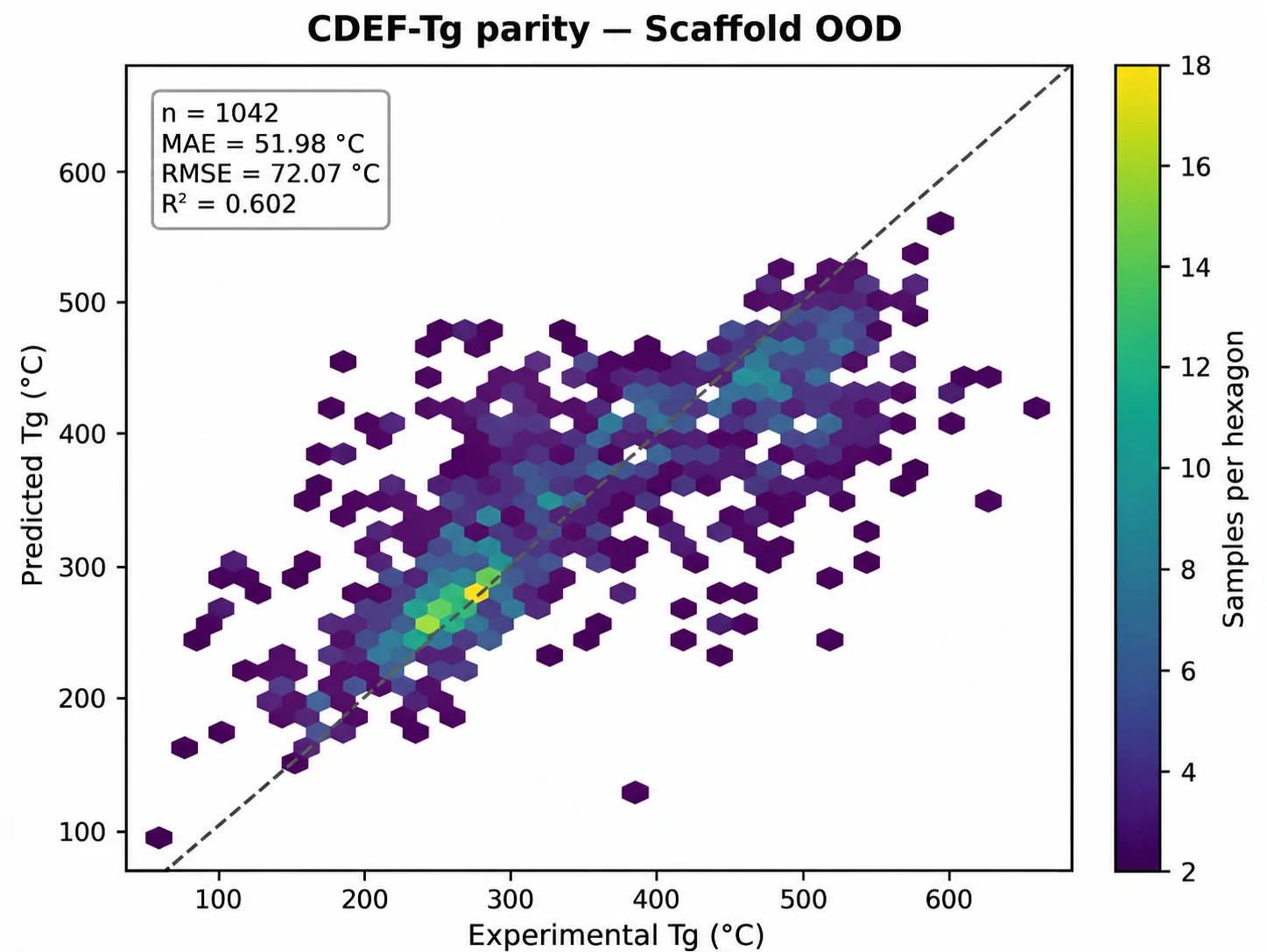
Recorded-versus-predicted Tg for CDEF-Tg under Scaffold OOD. Hexagonal-bin density shows the relationship between recorded and predicted Tg values for the scaffold-disjoint final-test partition (*n* = 1042). The dashed diagonal denotes the identity line. CDEF-Tg achieved MAE = 51.98 °C, RMSE = 72.07 °C, and R^2^ = 0.602 under this protocol. The axis label “Experimental Tg” denotes the Tg value recorded in the source dataset; its experimental provenance was not verified (Section 2.1).

Figure 5 shows the recorded-versus-predicted Tg relationship for CDEF-Tg under the scaffold-disjoint final-test partition (*n* = 1042), where CDEF-Tg achieved MAE = 51.98 °C, RMSE = 72.07 °C, and R^2^ = 0.602. This represents the highest MAE and RMSE, and the lowest R^2^, obtained by CDEF-Tg among the three locked evaluation protocols. As noted above, RDEF-Tg attained a marginally lower point-estimate MAE than CDEF-Tg under Scaffold OOD (51.67 °C versus 51.98 °C); CDEF-Tg did not achieve the lowest Scaffold-OOD MAE among the evaluated methods.

By the observed point estimates, Scaffold OOD was more challenging for CDEF-Tg than IID Group-Safe, with higher MAE (51.98 °C versus 47.81 °C) and RMSE (72.07 °C versus 67.34 °C) and lower R^2^ (0.602 versus 0.668). In contrast, Similarity OOD (T = 0.90) did not show a degradation relative to IID Group-Safe in the reported point estimates; MAE, RMSE, and R^2^ under Similarity OOD were each more favorable than under IID Group-Safe for CDEF-Tg. This pattern was consistent across the RDKit205-only, ChemBERTa384-only, and RDEF-Tg comparators as well (Table 3). Because an out-of-distribution protocol is defined by the chemical grouping and separation criterion applied to the data rather than by a guaranteed increase in numerical difficulty, these results do not support a claim that all out-of-distribution protocols were uniformly harder than the in-distribution protocol.

### 3.3. Bootstrap Evidence and Tg-Dependent Analysis

**Figure 6:**
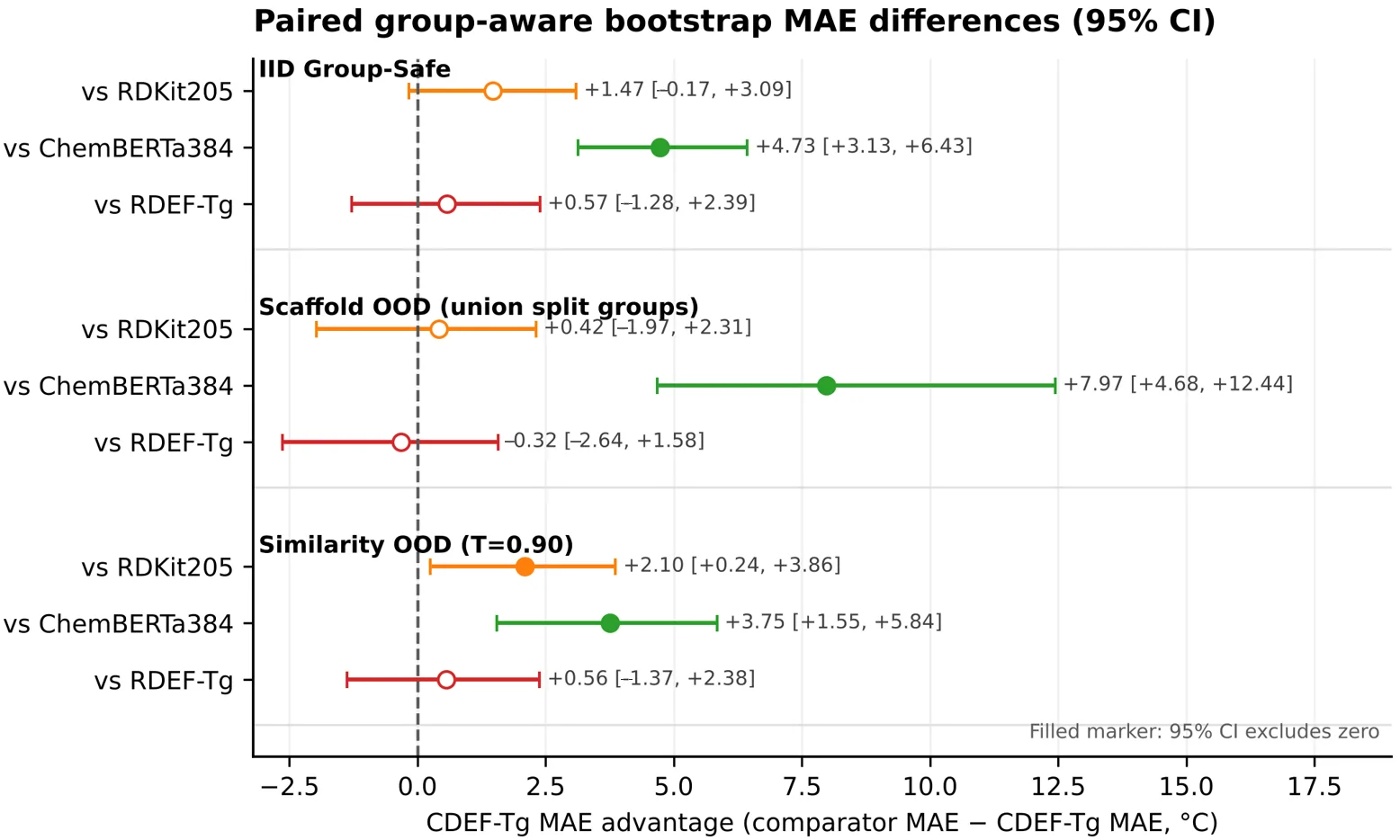
Paired group-aware bootstrap differences in MAE between CDEF-Tg and the frozen comparators under each locked final-test protocol (corrected group-aware analysis). Points denote the MAE advantage of CDEF-Tg, defined as comparator MAE minus CDEF-Tg MAE; positive values favor CDEF-Tg. Horizontal bars show 95% confidence intervals from 5000 paired bootstrap replicates that resample the actual split groups (union scaffold/fingerprint groups for Scaffold OOD). Filled markers indicate intervals that exclude zero. A macro summary is not plotted because macro intervals do not account for overlap among the test partitions (Table 4).

Uncertainty in the paired final-test comparisons was assessed with 5000 group-aware paired bootstrap replicates that resample the actual split groups of each protocol (Section 2.4; Table 4; Figure 6). Within IID Group-Safe, the CDEF-Tg MAE advantage over RDKit205-only (+1.47 [−0.17, +3.09] °C) and over RDEF-Tg (+0.57 [−1.28, +2.39] °C) included zero, whereas the advantage over ChemBERTa384-only did not (+4.73 [+3.13, +6.43] °C). Under Scaffold OOD, the MAE advantage over RDKit205-only was +0.42 [−1.97, +2.31] °C, and RDEF-Tg had a slightly lower point-estimate MAE than CDEF-Tg (−0.32 [−2.64, +1.58] °C); the Scaffold OOD RMSE and R^2^ intervals excluded zero against RDKit205-only (RMSE +3.74 [+1.29, +5.90] °C; R^2^ +0.042 [+0.014, +0.076]) and against RDEF-Tg (RMSE +3.05 [+0.47, +5.59] °C; R^2^ +0.034 [+0.005, +0.073]). Under Similarity OOD (T=0.90), the MAE advantage over RDKit205-only (+2.10 [+0.24, +3.86] °C) and over ChemBERTa384-only (+3.75 [+1.55, +5.84] °C) excluded zero, while the RDEF-Tg MAE interval included zero (+0.56 [−1.37, +2.38] °C).

Across the nine protocol–metric combinations per comparator, the intervals against ChemBERTa384-only excluded zero in all nine, those against RDKit205-only in three (Similarity OOD MAE; Scaffold OOD RMSE and R^2^), and those against RDEF-Tg in two (Scaffold OOD RMSE and R^2^). The macro paired MAE differences, the unweighted means of the protocol values and unchanged from the original analysis, were +1.33 °C (RDKit205-only), +5.49 °C (ChemBERTa384-only), and +0.27 °C (RDEF-Tg). Exploratory macro intervals from independent resampling of the protocols (with corrected Scaffold OOD groups) were [+0.19, +2.36], [+4.05, +7.20], and [−0.84, +1.35] °C, respectively; because they do not account for the overlap of test samples across protocols, they are not interpreted as dependence-adjusted evidence, and the earlier macro intervals are not retained for inferential interpretation.

Relative to RDEF-Tg, no protocol-level MAE interval excluded zero (Figure 6), and the Scaffold OOD point estimate favored RDEF-Tg; no MAE advantage of CDEF-Tg over RDEF-Tg is therefore supported, in line with the original conclusion. RMSE and R^2^ intervals against RDEF-Tg excluded zero only under Scaffold OOD (the two metrics are not independent, as noted above). Sign convention: positive values denote lower error (MAE, RMSE) or higher R^2^ for CDEF-Tg relative to the comparator.

**Figure 7:**
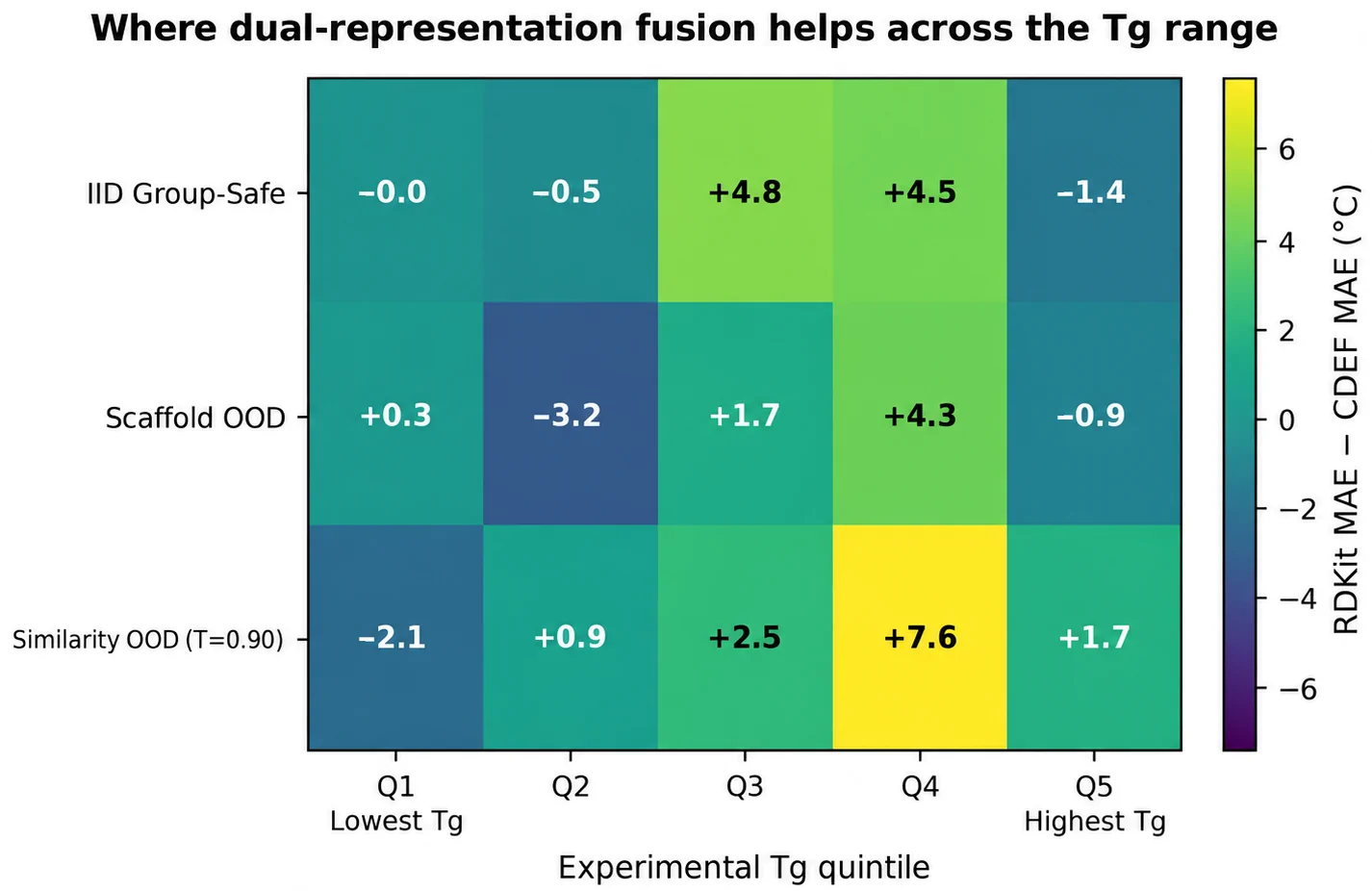
Tg-dependent benefit of descriptor–embedding fusion relative to the RDKit-only baseline. Each cell shows RDKit-only MAE minus CDEF-Tg MAE within a recorded-Tg quintile for a locked final-test protocol. Positive values indicate lower CDEF-Tg error, whereas negative values indicate lower RDKit-only error. The analysis is descriptive and illustrates how the observed benefit of fusion varies across the Tg range and evaluation protocols. Quintiles are defined on the Tg values recorded in the source dataset (labeled “Experimental Tg quintile” on the figure axis), whose experimental provenance was not verified (Section 2.1).

Figure 7 presents a descriptive analysis of the benefit of combining RDKit205 and ChemBERTa384 representations across the observed Tg range, defined as RDKit205-only MAE minus CDEF-Tg MAE within Tg quintiles of each locked final-test protocol, with positive values indicating lower CDEF-Tg error. This analysis indicates that the fusion benefit is not uniform across the Tg range or across protocols: within each of the three locked protocols, some Tg quintiles show a positive fusion benefit while others show a benefit close to zero or favoring the RDKit205-only representation. No consistent monotonic trend linking Tg magnitude to the size of the fusion benefit is evident across the three protocols. These observations are descriptive and do not support a mechanistic or causal explanation for where fusion is beneficial.

### 3.4. Control Experiments and Interpretability Analysis

Table 5 presents the four pre-specified model-selection candidates alongside additional control experiments evaluated on the validation partitions. The RDKit205 + MorganR2 control reached a macro validation MAE of 49.11 °C (RMSE 68.17 °C, R^2^ 0.651), about 4.04 °C lower than CDEF-Tg (53.15 °C), and it was lower in every protocol (IID Group-Safe 44.95 versus 50.26 °C; Scaffold OOD 55.19 versus 59.17 °C; Similarity OOD 47.18 versus 50.02 °C). CDEF-Tg therefore cannot be regarded as the best-performing representation among the alternatives examined. This control was not evaluated on the locked test partitions, because the test partitions were opened once under a single-evaluation rule, and any further test evaluation would be post hoc and not confirmatory. The RDKit205 + Random384 control gave a macro validation MAE of 56.77 °C, higher than both CDEF-Tg and the RDKit205-only baseline (54.82 °C); the CDEF-Tg gain was thus not reproduced by appending 384 uninformative features, but this does not show that ChemBERTa384 supplies information physically complementary to RDKit205, given that a Morgan-fingerprint partner performed better still.

The train-mean predictor gave test MAEs of 96.84, 97.21, and 98.33 °C for IID Group-Safe, Scaffold OOD, and Similarity OOD (T = 0.90), respectively (macro MAE 97.46 °C, macro RMSE 116.64 °C, macro R^2^ −0.011). The macro test MAE of CDEF-Tg (48.84 °C) is thus about half that of a constant predictor, while remaining large in absolute terms.

Block-level permutation importance for the refit CDEF-Tg model is summarized in Table 6. Permuting the RDKit205 block increased validation MAE by 43–51 °C (macro 48.23 °C) and permuting the ChemBERTa384 block by 13–18 °C (macro 15.57 °C), so the fitted model relies on both blocks, more strongly on RDKit205. This analysis does not demonstrate independent physical contributions of the blocks and does not quantify redundancy between them. The five highest-ranked RDKit205 columns by impurity-based importance were identical across the three protocols (zero-based indices 62, 37, 83, 36, and 204 in the locked RDKit205 order, corresponding to NumRotatableBonds, Kappa3, Phi, Kappa2, and qed, respectively, with NumRotatableBonds ranked first in each). Impurity-based importance indicates reliance of the fitted trees on these columns; it does not identify a physical mechanism, and no chemical interpretation is offered.

### 3.5. Split-Structure and Threshold Diagnostics

Table 7 summarizes the group structure of the three protocols and the nearest-neighbor similarity between each test sample and the development set. Singleton groups predominate in all three protocols, and the largest groups contain 15 (IID Group-Safe), 144 (Scaffold OOD), and 50 samples (Similarity OOD (T = 0.90)). The Scaffold OOD test partition comprises only 71 groups, one of which contains 144 samples (13.8% of the partition), so estimates under that protocol are inherently less stable. The median maximum similarity to the development set was 0.885 for IID Group-Safe, 0.775 for Scaffold OOD, and 0.851 for Similarity OOD; no Similarity OOD test sample had a development neighbor at or above 0.90, consistent with the zero cross-partition pairs at that threshold recorded at split construction, whereas 43.5% of IID Group-Safe and 10.8% of Scaffold OOD test samples did. Per-sample similarity values are available from the corresponding author on request. Relative to the dataset-wide groups, the test partitions contained larger groups on average: 1013 samples in 549 groups for IID Group-Safe (mean 1.85 versus 1.10 dataset-wide), 1042 in 71 for Scaffold OOD (14.7 versus 2.06), and 1022 in 266 for Similarity OOD (T = 0.90) (3.84 versus 1.27). The reason for this difference was not analyzed; the number of independent resampling units for the paired bootstrap is correspondingly small, particularly under Scaffold OOD.

Table 8 reports the Tanimoto-threshold connected-component audit on the identity-resolved dataset. At lower thresholds the graph chains into very large components: at 0.70 a single component contains 65.8% of all samples, and at 0.80 it contains 24.4%, both larger than the roughly 10% share of any held-out partition, whereas the largest component at 0.85 (337 samples, 3.4%) and at 0.90 (50 samples, 0.5%) is small enough for balanced group-aware partitioning. The audit reproduces the original cluster counts at 0.80 and 0.90 and supports 0.90 as a workable threshold; it does not show that 0.85 would have been unsuitable, and the prediction results at 0.70, 0.80, and 0.85 were not computed, so it is unknown whether the conclusions would remain unchanged at those thresholds.

### 3.6. Additional Baseline Evaluation and Robustness Analysis

Table 9 summarizes two additional validation-only analyses described in Section 2.5 neither was evaluated on the held-out test partitions or used for model selection. With LightGBM, adding ChemBERTa384 to RDKit205 lowered the validation MAE in all three protocols (IID Group-Safe 50.79 to 49.80 °C; Scaffold OOD 60.28 to 57.85 °C; Similarity OOD (T = 0.90) 51.84 to 50.03 °C), and the macro MAE, RMSE, and R^2^ changed from 54.31 to 52.56 °C, 72.00 to 70.09 °C, and 0.612 to 0.632, respectively. These are single-run point estimates, and no significance test was performed. The LightGBM models reached slightly lower macro validation MAE than the corresponding frozen ExtraTrees entries (52.56 versus 53.15 °C for the fused representation and 54.31 versus 54.82 °C for RDKit205); since LightGBM was run in one configuration after the original selection, this difference is descriptive and does not change the frozen CDEF-Tg model or the locked test evaluation. The RDKit205 + MorganR2 ExtraTrees control (49.11 °C; Table 5) still had the lowest macro validation MAE among the entries reported in this article.

Across the five ExtraTrees random states, the macro validation MAE was 53.06 ± 0.10 °C for CDEF-Tg and 54.77 ± 0.13 °C for RDKit205, and CDEF-Tg had lower MAE, lower RMSE, and higher R^2^ than RDKit205 in each of the 15 random-state–protocol combinations. The per-protocol MAE differences averaged 1.30 (IID Group-Safe), 0.67 (Scaffold OOD), and 3.15 °C (Similarity OOD (T = 0.90)), with a smallest single difference of 0.22 °C under Scaffold OOD, and the largest per-protocol standard deviation was 0.33 °C. The direction of the CDEF-Tg versus RDKit205 comparison was therefore stable with respect to the random state of the model on these fixed partitions. This is not split-seed robustness and does not extend to the held-out test sets, and the comparison is descriptive: no hypothesis test was applied, and the test-set bootstrap intervals in Section 3.3 remain the inferential evidence for the locked comparison. The random-state 42 rerun differed slightly from the frozen Stage-04B values (Section 2.5), which are retained in Table 2 and Table 5.

## 4. Discussion

Within the evaluated dataset and protocols, adding the frozen ChemBERTa384 embedding to the RDKit205 descriptors produced lower point-estimate error and higher explained variance than either representation alone in every locked final-test protocol. The size and certainty of this benefit are, however, modest and uneven. Against the ChemBERTa384-only model, the advantage was distinguishable from zero in every protocol and metric, whereas against the RDKit205-only model the macro MAE point estimate was +1.33 °C and the protocol-level paired intervals excluded zero only for MAE under Similarity OOD and for RMSE under Scaffold OOD (the R^2^ interval was concordant with RMSE and is not independent evidence, since both metrics are computed from the same resampled replicates). Because the three test partitions overlap and the original macro analysis resampled them independently, macro intervals are not interpreted as inferential evidence. The additional control experiments further limit what can be concluded: an RDKit205 + MorganR2 concatenation achieved a lower macro validation MAE than CDEF-Tg (49.11 versus 53.15 °C), so ChemBERTa384 is not shown to be the preferable partner for RDKit205, and the RDKit205 + Random384 control (56.77 °C) indicates only that the CDEF-Tg gain was not reproduced by appending 384 uninformative features. One plausible reading is that fixed descriptors and learned or fingerprint-based structural encodings carry partly non-redundant information about glass-transition behavior [25,31]; this is consistent with the data but is not demonstrated, because redundancy between feature blocks was not quantified and the block-permutation results (Table 6) show only that the fitted model relies on both blocks, more strongly on RDKit205. The finding is therefore best stated as an observation about predictive performance in a polyimide-oriented dataset, not as an argument that descriptor–embedding fusion should be preferred for polymer property prediction [1,2]. Additional validation-only analyses (Table 9) were consistent in direction with this interpretation without strengthening it: adding ChemBERTa384 to RDKit205 lowered the validation MAE of a single additional LightGBM comparator configuration in all three protocols (this comparator is distinct from CDEF-Tg, which is defined by the ExtraTreesRegressor frozen before the final test, and it did not replace or alter the frozen model or any locked result), and the CDEF-Tg versus RDKit205 ordering was stable across five ExtraTrees random states on fixed partitions. These results are descriptive point estimates, no significance test was applied, and they do not address split-seed variability or comparison with graph-based or fine-tuned models.

Previous work has established descriptor- and fingerprint-based prediction of polymer Tg [1,2,18], graph-based Tg models [21,22], polymer and molecular language representations [26,27,28], transfer-learning strategies for data-scarce settings [24], and multimodal representation fusion in molecular property prediction [32,33]. The present study is positioned relative to these lines of work rather than as a replacement for them. Its distinguishing features are compact early fusion of RDKit205 and frozen ChemBERTa384, compared directly against both constituent single-representation models and against RDEF-Tg as an alternative fusion strategy; validation-only model selection, with the architecture frozen before final-test evaluation; evaluation under IID Group-Safe, Scaffold OOD, and Similarity OOD (T = 0.90), in line with the motivation for chemically informed validation [36,37]; and paired group-aware bootstrap evidence reported alongside point estimates. As discussed below, the ordering of the two fusion strategies is metric- and protocol-dependent. This study also does not establish superiority over the graph-based, language-model, or transfer-learning approaches cited above, which were not evaluated under the present protocols. The methodological novelty of CDEF-Tg is limited: it applies standard early concatenation and a standard tree ensemble to two existing representations. The contribution claimed here is the controlled, leakage-aware evaluation of that combination, including the reporting of results that qualify it, rather than a new learning algorithm or state-of-the-art accuracy.

The comparison between CDEF-Tg and RDEF-Tg is metric- and protocol-dependent. No protocol-level MAE interval for this comparison excluded zero, and RDEF-Tg had the slightly lower Scaffold OOD MAE point estimate (51.67 versus 51.98 °C), so no MAE advantage of CDEF-Tg over RDEF-Tg is supported. RMSE and R^2^ intervals excluded zero only under Scaffold OOD. RMSE weights large residuals more heavily than MAE, so the divergence between metrics indicates that the two models differ in how they distribute large errors; the present analysis did not characterize those error distributions. Early concatenation is architecturally simpler than the residual-fusion pipeline, which is an observation about architecture and not a measured advantage in computational cost or interpretability, neither of which was evaluated.

The three evaluation protocols probe distinct forms of structural generalization, and the differences among them are more informative than the macro summary alone. For CDEF-Tg, the Scaffold OOD point estimates were less favorable than those under IID Group-Safe, consistent with scaffold-level separation presenting a harder generalization problem for this model and dataset. Similarity OOD (T = 0.90) did not show the corresponding degradation relative to IID Group-Safe. This asymmetry reflects a general property of out-of-distribution evaluation that deserves emphasis: an OOD designation records the structural separation criterion applied during partitioning, not a guaranteed increase in numerical difficulty. Murcko-scaffold grouping and connected-component grouping at a Tanimoto threshold of 0.90 divide the chemical space along different axes, and the distributional shift each induces for a given model and dataset need not be comparable in magnitude. Nothing in these results supports the broader claim that OOD splits are inherently harder than in-distribution evaluation; the practical difficulty of a grouping criterion is protocol-specific and must be established empirically for each criterion considered [34,36,37]. The corollary for method development is that a single OOD definition provides limited assurance about generalization, since a model may appear stable under one separation criterion and degrade under another. For Similarity OOD, the nearest-neighbor analysis (Table 7) offers a descriptive explanation of why it need not be harder than IID: the protocol removes only development neighbors at Tanimoto similarity ≥ 0.90, and 80.5% of its test samples still had a development neighbor at ≥0.80, comparable to IID Group-Safe (82.2%) and well above Scaffold OOD (42.4%). Differences in Tg composition between partitions and sampling variability may also contribute. However, the sampling variability of the absolute MAE of each protocol was not estimated, and these explanations were not tested separately.

These comparisons rest on an evaluation design intended to limit the influence of incidental train–test similarity on reported performance [34,35,36]. Identity resolution of the underlying records, group-aware assignment with Tg rank-decile balancing, and the use of three distinct structural-generalization regimes together reduce the risk that apparent predictive capability reflects near-duplicate chemistry shared across partitions. Model selection was conducted using validation evidence alone, and CDEF-Tg was frozen before final-test evaluation; following the freeze, a single locked final-test evaluation was performed, with no subsequent model selection, feature selection, hyperparameter tuning, architecture replacement, or result-driven retraining. Paired group-aware bootstrap resampling supplied interval evidence alongside point estimates, which matters because a single held-out point estimate conveys little about whether an observed difference between closely performing models would persist under resampling. The distinction is illustrated by the differing MAE and RMSE evidence in the RDEF-Tg comparison. These procedures reduce, but do not eliminate, possible sources of evaluation bias. They do not address heterogeneity or systematic error in how the underlying Tg values were originally obtained and curated, and the study employed a single fixed split seed (42); ExtraTrees random-state sensitivity was examined only on the fixed validation partitions (five random states; Table 9), and repeated splits with different split seeds were not performed, so the sensitivity of the reported comparisons to alternative split seeds, alternative grouping definitions, or the global Tg-decile balancing is unknown rather than assessed. The three test partitions also share samples and are therefore not independent. A random-state 42 rerun of the ExtraTrees models on the fixed validation partitions did not exactly reproduce the frozen Stage-04B validation results (macro MAE differences of 0.050 °C for RDKit205 and 0.009 °C for CDEF-Tg; per-protocol differences up to 0.25 °C); the origin of these small differences was not identified, the frozen Stage-04B and locked Stage-05 results were retained unchanged, and no claim of exact reproduction is made.

The Tg-quintile analysis indicates that the difference between RDKit205-only MAE and CDEF-Tg MAE varied across quintiles of observed Tg and across evaluation protocols, without a consistent monotonic pattern across the range. Certain quintile–protocol combinations showed a small or negative difference, others a substantially larger positive one. This is consistent with the fusion-associated benefit being context-dependent within the observed Tg range rather than uniform across it. The analysis remains descriptive in character: it does not identify which chemical or thermal characteristics distinguish quintiles with larger from those with smaller differences, and it supports no causal or mechanistic account of the variation.

Several boundaries on the present evidence warrant explicit statement. The evaluation is confined to a single identity-resolved polymer Tg dataset, so the reported performance is conditioned on the chemical coverage and labeling conventions of that dataset. Both representations are derived entirely from SMILES strings, and the framework incorporates no explicit morphological descriptors, molecular-weight distribution, processing-history variables, or experimental measurement conditions, each of which can influence experimentally reported Tg [3,4,8,45]. The ChemBERTa384 embedding was used in frozen form rather than fine-tuned for this task, and the study did not evaluate whether task-specific fine-tuning of ChemBERTa would alter predictive performance. The principal fusion estimator belongs to a single model family, and the evidence does not extend to estimators with materially different inductive biases. Extrapolation beyond the chemical space represented in the evaluated partitions should be treated as unverified, and the Tg-quintile analysis should be read descriptively rather than explanatorily. The dataset is polyimide-oriented, and its records carry no references, measurement methods, or thermal-history information. Experimentally measured Tg depends strongly on heating or cooling rate and sample history [3,4]; the recorded values reflect unspecified conditions, and fusing two SMILES-derived representations cannot compensate for this, so predictions should be read as estimates of the dataset-recorded Tg. No numerical noise floor could be established, although the single duplicate pair differed by 59.1 °C, and the 119 records at or above 600 °C were not independently verified; CDEF-Tg MAE was largest in the highest Tg quintile in every protocol (80.2, 78.3, and 81.2 °C for IID Group-Safe, Scaffold OOD, and Similarity OOD, versus at most 57.9 °C in the lower quintiles), which is consistent with, but does not demonstrate, greater label noise or sparser coverage in that range. A single LightGBM configuration was evaluated on the validation partitions only (Table 9), without a documented hyperparameter search, significance test, or test-set evaluation; tuned gradient-boosting models, graph neural networks, fine-tuned ChemBERTa, and polyBERT were not evaluated, so no statement is made about relative performance against those families or about state-of-the-art status. An MAE of about 49 °C is too large for fine-grained design decisions, and no comparison with literature models on a comparable dataset and protocol was made.

Within these boundaries, a few considerations follow. Adding frozen ChemBERTa384 embeddings to RDKit205 descriptors improved predictive performance relative to either representation alone in this evaluation, although the gain over RDKit205 alone was small and not consistently distinguishable at the protocol level, and the Morgan-fingerprint control shows that alternative representation choices should be compared on equal terms. Structural generalization is better assessed under more than one leakage-safe split definition, since behavior under one separation criterion did not mirror behavior under another. Reporting paired interval evidence at the protocol level, with resampling units that match the split groups, conveys information that point estimates alone do not, and macro summaries over overlapping test partitions are best presented descriptively. A macro MAE of about 49 °C is roughly half that of a train-mean predictor (97.46 °C) but large in absolute terms; the framework is best regarded as a controlled benchmark of representation choices rather than a deployable predictor. All of these considerations are specific to the dataset and evaluation framework examined here and should be re-established on any new dataset or protocol before being relied upon.

## 5. Conclusions

This study introduced CDEF-Tg, a compact fusion framework that combines RDKit205 physicochemical descriptors with frozen ChemBERTa384 molecular-language embeddings through early feature concatenation and an ExtraTreesRegressor, and evaluated it on a polyimide-oriented dataset under three leakage-safe protocols—IID Group-Safe, Scaffold OOD, and Similarity OOD (T = 0.90). The architecture was selected using validation-only evidence and frozen before a single locked final-test evaluation. Across all three protocols, CDEF-Tg achieved lower point-estimate MAE and RMSE and higher R^2^ than either single-representation ablation (macro MAE 48.84 °C versus 50.17 °C for RDKit205-only and 54.33 °C for ChemBERTa384-only; 97.46 °C for a train-mean predictor). Corrected paired bootstrap analyses using the actual split groups distinguished CDEF-Tg from the ChemBERTa384-only model in every protocol, but distinguished it from the RDKit205-only model only in MAE under Similarity OOD and in RMSE (with concordant R^2^) under Scaffold OOD, and showed no MAE advantage over the residual-fusion alternative RDEF-Tg, which also had a marginally lower Scaffold OOD MAE point estimate. Additional validation-only controls showed that an RDKit205 + Morgan-fingerprint concatenation achieved a lower macro validation MAE than CDEF-Tg, so CDEF-Tg should not be regarded as uniformly superior or as the best representation among those examined. Similarity OOD did not exhibit degradation relative to IID in the point estimates, in contrast to Scaffold OOD, indicating that OOD definitions grounded in different chemical relations need not yield a uniform ordering of difficulty. Taken together, the results support a modest, protocol-dependent benefit of adding frozen ChemBERTa384 embeddings to RDKit205 descriptors within this dataset, without establishing a mechanistic basis for complementarity, a state-of-the-art claim, or generalization to other polymer classes. Additional validation-only analyses (a LightGBM baseline and five ExtraTrees random states on fixed splits) were consistent in direction with the benefit of adding ChemBERTa384 to RDKit205 but are descriptive and were not subjected to significance testing and do not establish split-seed robustness; evaluation across different split seeds, graph-based and fine-tuned model families, and datasets with documented measurement conditions remain to be examined.

## Figures and Tables

**Table 1:** Dataset and leakage-safe evaluation protocols. Train, validation, and locked test sizes are reported for IID Group-Safe, Scaffold OOD, and Similarity OOD (T = 0.90). Splits were generated with group-aware assignment using Tg rank-decile balancing. The grouping unit differs by protocol to prevent leakage of the chemical relation targeted by each evaluation setting. Validation was reserved for model-selection decisions; the test partitions were reserved for final evaluation.

Protocol	Train *n*	Validation *n*	Test *n*	Atomic Grouping Unit	Evaluation Purpose	Test Tg Range (°C)
IID Group-Safe	8038	1014	1013	Exact Morgan FP group (r = 2, 2048-bit, chirality-aware hash)	primary in-distribution comparison while preventing identical fingerprint input leakage	20.00–678.00
Scaffold OOD	7990	1033	1042	Union of non-empty Murcko scaffold and exact Morgan FP group	scaffold-level chemical generalization	61.00–659.00
Similarity OOD (T = 0.90)	8021	1022	1022	Morgan/Tanimoto ≥ 0.90 connected component	local-neighborhood chemical OOD evaluation	20.00–643.89

All splits were fixed with seed 42 using group-aware assignment and 10 global Tg rank-decile bins. Validation was reserved for model/hyperparameter decisions; the test partition remained locked until one-shot final evaluation.

**Table 2:** Validation benchmark of compact frozen baselines. Validation-only MAE, RMSE, and R^2^ are reported for ExtraTrees models using RDKit205, Morgan radius-2 fingerprints, or ChemBERTa384 representations, together with an RBF-SVR ChemBERTa baseline. These results were obtained before final-test opening and provide the baseline evidence used during method development.

Protocol	Model	Representation	Train *n*	Validation *n*	MAE (°C)	RMSE (°C)	R^2^
IID Group-Safe	ExtraTrees + RDKit205	RDKit205	8038	1014	51.511	71.408	0.6384
IID Group-Safe	ExtraTrees + MorganR2	MorganR2 (2048-bit)	8038	1014	52.655	75.167	0.5994
IID Group-Safe	ExtraTrees + ChemBERTa384	ChemBERTa384	8038	1014	54.418	71.891	0.6335
IID Group-Safe	SVR-RBF + ChemBERTa384	ChemBERTa384	8038	1014	58.631	78.242	0.5659
Scaffold OOD	ExtraTrees + RDKit205	RDKit205	7990	1033	59.873	77.813	0.5167
Scaffold OOD	ExtraTrees + MorganR2	MorganR2 (2048-bit)	7990	1033	65.149	86.273	0.4059
Scaffold OOD	ExtraTrees + ChemBERTa384	ChemBERTa384	7990	1033	63.059	78.645	0.5063
Scaffold OOD	SVR-RBF + ChemBERTa384	ChemBERTa384	7990	1033	64.647	80.944	0.477
Similarity OOD (T = 0.90)	ExtraTrees + RDKit205	RDKit205	8021	1022	53.084	74.414	0.6001
Similarity OOD (T = 0.90)	ExtraTrees + MorganR2	MorganR2 (2048-bit)	8021	1022	54.182	79.32	0.5456
Similarity OOD (T = 0.90)	ExtraTrees + ChemBERTa384	ChemBERTa384	8021	1022	55.352	75.125	0.5924
Similarity OOD (T = 0.90)	SVR-RBF + ChemBERTa384	ChemBERTa384	8021	1022	57.392	78.751	0.5521

All values are validation-only results; final-test information was not used for baseline comparison or model selection. Lower MAE/RMSE and higher R^2^ indicate better predictive performance.

**Table 3:** Locked final-test performance and minimal ablation. Final-test MAE, RMSE, and R^2^ are reported for the frozen CDEF-Tg proposed model, RDKit205-only and ChemBERTa384-only ExtraTrees ablations, and the RDEF-Tg fusion-strategy ablation under all three locked evaluation protocols. CDEF-Tg was selected and frozen before the final-test sets were opened; no post-test tuning or model replacement was performed. Errors are computed against the Tg values recorded in the source dataset [39]; per-record literature references and measurement conditions are not available in that dataset.

Protocol	Model	Role	MAE (°C)	RMSE (°C)	R^2^	λ
IID Group-Safe	CDEF-Tg	Proposed model	47.808	67.34	0.668	
IID Group-Safe	ExtraTrees + RDKit205	Descriptor-only ablation	49.276	69.883	0.6425	
IID Group-Safe	ExtraTrees + ChemBERTa384	Embedding-only ablation	52.54	72.699	0.6131	
IID Group-Safe	RDEF-Tg	Fusion-strategy ablation	48.378	69.037	0.6511	1.000
Scaffold OOD	CDEF-Tg	Proposed model	51.981	72.073	0.6016	
Scaffold OOD	ExtraTrees + RDKit205	Descriptor-only ablation	52.401	75.811	0.5592	
Scaffold OOD	ExtraTrees + ChemBERTa384	Embedding-only ablation	59.955	78.917	0.5223	
Scaffold OOD	RDEF-Tg	Fusion-strategy ablation	51.666	75.121	0.5671	0.770
Similarity OOD (T = 0.90)	CDEF-Tg	Proposed model	46.736	65.824	0.6836	
Similarity OOD (T = 0.90)	ExtraTrees + RDKit205	Descriptor-only ablation	48.836	68.922	0.6531	
Similarity OOD (T = 0.90)	ExtraTrees + ChemBERTa384	Embedding-only ablation	50.49	69.48	0.6475	
Similarity OOD (T = 0.90)	RDEF-Tg	Fusion-strategy ablation	47.297	67.472	0.6676	1.000

CDEF-Tg was frozen before final-test opening. No post-test tuning, model replacement, or hyperparameter search was permitted. λ applies only to the RDEF-Tg residual-fusion ablation.

**Table 4:** Paired group-aware bootstrap evidence for CDEF-Tg relative to frozen comparators (corrected group-aware analysis). Entries are the CDEF-Tg advantage (comparator minus CDEF-Tg for MAE and RMSE; CDEF-Tg minus comparator for R^2^) with 95% confidence intervals from 5000 paired bootstrap replicates that resample the actual split groups of each protocol. Macro rows give the unweighted mean of the protocol-level point estimates; their intervals (†) come from independent resampling of the protocols, do not account for the overlap of test samples across protocols, and are exploratory.

Comparator	Protocol	ΔMAE [95% CI] (°C)	ΔRMSE [95% CI] (°C)	ΔR^2^ [95% CI]
ExtraTrees + RDKit205	IID Group-Safe	+1.47 [−0.17, +3.09]	+2.54 [−0.11, +5.08]	+0.026 [−0.001, +0.053]
ExtraTrees + RDKit205	Scaffold OOD	+0.42 [−1.97, +2.31]	+3.74 [+1.29, +5.90]	+0.042 [+0.014, +0.076]
ExtraTrees + RDKit205	Similarity OOD (T = 0.90)	+2.10 [+0.24, +3.86]	+3.10 [−0.34, +6.07]	+0.030 [−0.003, +0.063]
ExtraTrees + RDKit205	Macro (exploratory)	+1.33 [+0.19, +2.36] †	+3.13 [+1.45, +4.66] †	+0.033 [+0.016, +0.051] †
ExtraTrees + ChemBERTa384	IID Group-Safe	+4.73 [+3.13, +6.43]	+5.36 [+3.30, +7.51]	+0.055 [+0.034, +0.077]
ExtraTrees + ChemBERTa384	Scaffold OOD	+7.97 [+4.68, +12.44]	+6.84 [+4.06, +10.64]	+0.079 [+0.049, +0.129]
ExtraTrees + ChemBERTa384	Similarity OOD (T = 0.90)	+3.75 [+1.55, +5.84]	+3.66 [+0.17, +6.44]	+0.036 [+0.002, +0.063]
ExtraTrees + ChemBERTa384	Macro (exploratory)	+5.49 [+4.05, +7.20] †	+5.29 [+3.66, +7.01] †	+0.057 [+0.040, +0.077] †
RDEF-Tg	IID Group-Safe	+0.57 [−1.28, +2.39]	+1.70 [−1.18, +4.49]	+0.017 [−0.011, +0.047]
RDEF-Tg	Scaffold OOD	−0.32 [−2.64, +1.58]	+3.05 [+0.47, +5.59]	+0.034 [+0.005, +0.073]
RDEF-Tg	Similarity OOD (T = 0.90)	+0.56 [−1.37, +2.38]	+1.65 [−1.46, +4.34]	+0.016 [−0.015, +0.045]
RDEF-Tg	Macro (exploratory)	+0.27 [−0.84, +1.35] †	+2.13 [+0.45, +3.68] †	+0.022 [+0.005, +0.041] †

Positive differences indicate better CDEF-Tg performance. Protocol-level intervals resample the actual split groups (atomic group identifier; union scaffold/fingerprint groups for Scaffold OOD). † Exploratory intervals from independently resampled protocols; not dependence-adjusted. No protocol-level MAE interval against RDEF-Tg excludes zero; no MAE advantage over RDEF-Tg is claimed.

**Table 5:** Validation-only comparison of the pre-specified model-selection candidates and additional control experiments. MAE is given per protocol and as the macro mean; macro RMSE and R^2^ are also shown. The additional controls were not used in the original model selection.

Origin	Model	IID MAE	Scaffold MAE	Similarity MAE	Macro MAE	Macro RMSE	Macro R^2^
Model selection	CDEF-Tg (EarlyFusion ExtraTrees)	50.26	59.17	50.02	53.15	70.41	0.629
Model selection	RDEF-Tg	50.15	59.63	51.82	53.87	73.63	0.595
Model selection	ResidualFusion_Lambda1	50.15	59.67	51.82	53.88	73.64	0.595
Model selection	ExtraTrees + RDKit205 (frozen)	51.51	59.87	53.08	54.82	74.55	0.585
Additional analysis	RDKit205 + MorganR2 (2253-D)	44.95	55.19	47.18	49.11	68.17	0.651
Additional analysis	RDKit205 + Random384 (589-D)	54.60	61.69	54.04	56.77	74.19	0.589
Additional analysis	Train-mean predictor	98.42	93.83	98.18	96.81	116.55	−0.008

All values are validation-only. The pre-specified candidate results were used for model selection before final-test evaluation. The additional controls were evaluated subsequently and did not influence model selection or the frozen configuration. Random384 denotes 384 synthetic Gaussian features whose per-dimension mean and standard deviation were taken from the training-partition ChemBERTa384 embeddings (Section 2.5). MAE and RMSE are in °C.

**Table 6:** Block-level permutation importance of the refit CDEF-Tg model on the validation partitions. Entries are the mean increase in MAE over three permutation repeats (range in parentheses).

Protocol	Unpermuted Validation MAE (°C)	ΔMAE, RDKit205 Block Permuted (°C)	ΔMAE, ChemBERTa384 Block Permuted (°C)
IID Group-Safe	50.26	51.30 (49.76–54.01)	18.05 (17.86–18.25)
Scaffold OOD	59.17	43.42 (43.03–44.16)	12.61 (12.47–12.85)
Similarity OOD (T = 0.90)	50.02	49.98 (49.23–51.23)	16.04 (15.56–16.82)
Macro	53.15	48.23	15.57

Validation-only analysis. The increase in error reflects reliance of the fitted model on each block, not an independent causal contribution or a measure of redundancy between blocks.

**Table 7:** Split-structure diagnostics for the three protocols. Group counts refer to the whole identity-resolved dataset (10,065 records); similarity statistics refer to the maximum Morgan Tanimoto similarity between each test sample and the development (training plus validation) set.

Diagnostic	IID Group-Safe	Scaffold OOD	Similarity OOD (T = 0.90)
Groups (whole dataset)	9127	4883	7910
Largest group (samples)	15	144	50
Singleton groups, *n* (%)	8585 (94.1%)	3725 (76.3%)	6949 (87.9%)
Mean group size	1.10	2.06	1.27
Groups in the test partition	549	71	266
Max. similarity to development set: median (5th–95th percentile)	0.885 (0.681–0.976)	0.775 (0.567–0.929)	0.851 (0.702–0.895)
Test samples with max. similarity ≥ 0.80	82.2%	42.4%	80.5%
Test samples with max. similarity ≥ 0.90	43.5%	10.8%	0.0%

Computed post hoc from the frozen split manifests with RDKit (Morgan radius 2, 2048 bits, chirality-aware). The three test partitions share 129 (IID/Scaffold), 255 (IID/Similarity), and 75 (Scaffold/Similarity) samples, and 4 samples in all three.

**Table 8:** Tanimoto-threshold connected-component analysis (Morgan radius 2, 2048-bit, chirality-aware). The audit characterizes grouping structure only; no additional predictive-performance experiments were conducted at any threshold, and the original Similarity OOD (T = 0.90) evaluation is unchanged.

Threshold	Clusters	Largest Cluster (Samples, % of Dataset)	Singleton Clusters	Pairs at or Above Threshold
0.70	1835	6624 (65.81%)	1495	87,177
0.80	4399	2460 (24.44%)	3623	22,996
0.85	6447	337 (3.35%)	5481	10,675
0.90	7910	50 (0.50%)	6949	5245

Cluster counts at 0.80 (4399) and 0.90 (7910) match the values recorded at split construction.

**Table 9:** Additional validation-only results of the LightGBM baseline and of ExtraTrees across five random states on the fixed validation partitions. Validation MAE is given per protocol and as the unweighted macro mean, together with macro RMSE and R^2^. MAE and RMSE are in °C.

Model	IID Group-Safe MAE	Scaffold OOD MAE	Similarity OOD MAE	Macro MAE	Macro RMSE	Macro R^2^
LightGBM + RDKit205 (single run)	50.79	60.28	51.84	54.31	72.00	0.612
LightGBM + RDKit205 + ChemBERTa384 (single run)	49.80	57.85	50.03	52.56	70.09	0.632
ExtraTrees + RDKit205 (5 random states, mean ± SD)	51.52 ± 0.12	59.65 ± 0.33	53.15 ± 0.05	54.77 ± 0.13	74.43 ± 0.18	0.587 ± 0.0020
ExtraTrees + RDKit205 + ChemBERTa384 (CDEF-Tg) (5 random states, mean ± SD)	50.22 ± 0.15	58.97 ± 0.16	50.00 ± 0.13	53.06 ± 0.10	70.27 ± 0.06	0.631 ± 0.0005

LightGBM: one configuration, random_state = 42. ExtraTrees: random states 42, 100, 200, 300, and 400 on the fixed split (seed 42); ± is the sample standard deviation over random states, not a confidence interval. All values are from additional validation-only analyses; no significance tests were performed, and the split seed was not varied. The frozen Stage-04B validation values (Table 2 and Table 5) are unchanged.

## Data Availability

The raw glass transition temperature dataset used in this study (PI_Tg_10066.csv) is publicly available in the UniPolymer GitHub repository (hjunquan1100/UniPolymer, path: pi_forward_prediction/data/PI_Tg_10066.csv), accessible directly at https://github.com/hjunquan1100/UniPolymer (accessed on 10 September 2026), associated with Hu et al. (2026) [39]. The identity-resolved dataset analyzed in the present study was derived from this publicly available source. The leakage-safe split manifests and processed analysis artifacts generated during the present study are available from the corresponding author upon reasonable request. The source file contains only SMILES strings and Tg values, without reference or measurement-method annotations. Per-sample similarity values, the additional control and permutation outputs, and the corrected bootstrap tables are available from the corresponding author upon reasonable request.

## Abbreviations

The following abbreviations are used in this manuscript:

CDEF-Tg	Compact Descriptor–Embedding Fusion for Glass Transition Temperature Prediction
CI	Confidence interval
ChemBERTa384	384-dimensional ChemBERTa molecular embedding
IID	Independent and identically distributed
MAE	Mean absolute error
ML	Machine learning
OOD	Out-of-distribution
QSPR	Quantitative structure–property relationship
RBF	Radial basis function
RDEF-Tg	Residual Descriptor–Embedding Fusion for Glass Transition Temperature Prediction
RDKit205	205-dimensional RDKit physicochemical descriptor representation
RMSE	Root mean squared error
R^2^	Coefficient of determination
SMILES	Simplified Molecular Input Line Entry System
SVR	Support vector regression
Tg	Glass transition temperature
